# The histone modification H3 lysine 27 tri-methylation has conserved gene regulatory roles in the triplicated genome of *Brassica rapa* L.

**DOI:** 10.1093/dnares/dsz021

**Published:** 2019-10-17

**Authors:** Ayasha Akter, Satoshi Takahashi, Weiwei Deng, Daniel J Shea, Etsuko Itabashi, Motoki Shimizu, Naomi Miyaji, Kenji Osabe, Namiko Nishida, Yutaka Suzuki, Chris A Helliwell, Motoaki Seki, William James Peacock, Elizabeth S Dennis, Ryo Fujimoto

**Affiliations:** 1 Graduate School of Agricultural Science, Kobe University, Kobe, Japan; 2 Center for Sustainable Resource Science, RIKEN, Yokohama, Kanagawa, Japan; 3 Centre for Crop and Disease Management (CCDM), School of Molecular and Life Sciences, Curtin University, Perth, WA, Australia; 4 Graduate School of Science and Technology, Niigata University, Niigata, Japan; 5 Department of Genomics and Breeding, Iwate Biotechnology Research Center, Narita, Kitakami, Iwate, Japan; 6 Plant Epigenetics Unit, Okinawa Institute of Science and Technology Graduate University, Onna-son, Okinawa, Japan; 7 Department of Computational Biology, Graduate School of Frontier Sciences, The University of Tokyo, Kashiwa, Chiba, Japan; 8 Agriculture and Food, CSIRO, Canberra, ACT, Australia; 9 Cluster for Pioneering Research, RIKEN, 2-1 Hirosawa, Wako, Saitama, Japan; 10 Kihara Institute for Biological Research, Yokohama City University, Yokohama, Kanagawa, Japan; 11 Department of Life Sciences, University of Technology, Sydney, Broadway, NSW, Australia

**Keywords:** histone H3 lysine 27 tri-methylation, vernalization, epigenetics, Brassica, FLOWERING LOCUS C

## Abstract

*Brassica rapa* L. is an important vegetable and oilseed crop. We investigated the distribution of the histone mark tri-methylation of H3K27 (H3K27me3) in *B. rapa* and its role in the control of gene expression at two stages of development (2-day cotyledons and 14-day leaves) and among paralogs in the triplicated genome. H3K27me3 has a similar distribution in two inbred lines, while there was variation of H3K27me3 sites between tissues. Sites that are specific to 2-day cotyledons have increased transcriptional activity, and low levels of H3K27me3 in the gene body region. In 14-day leaves, levels of H3K27me3 were associated with decreased gene expression. In the triplicated genome, H3K27me3 is associated with paralogs that have tissue-specific expression. Even though *B. rapa* and *Arabidopsis thaliana* are not closely related within the Brassicaceae, there is conservation of H3K27me3-marked sites in the two species. Both *B. rapa* and *A. thaliana* require vernalization for floral initiation with *FLC* being the major controlling locus. In all four *BrFLC* paralogs, low-temperature treatment increases H3K27me3 at the proximal nucleation site reducing *BrFLC* expression. Following return to normal temperature growth conditions, H3K27me3 spreads along all four *BrFLC* paralogs providing stable repression of the gene.

## 1. Introduction

*Brassica rapa* L. encompasses commercially important vegetable crops including Chinese cabbage (var. *pekinensis*), pak choi (var. *chinensis*), and turnip (var. *rapa*) as well as oilseed crops (var. *oleifera*). The *B. rapa* genome has undergone a whole-genome triplication resulting in multiple copies of paralogous genes. Three subgenomes, the least fractioned (LF) subgenome and two more fractionated subgenomes (MF1 and MF2), are recognized within the *B. rapa* genome.^1^

Activity levels of the component genes of the genomes are regulated by transcription factors and epigenetic modifications. The regulation of gene expression by epigenetic modification is crucial for the development and adaptation of plants to changing environments.^2^^,^^3^ Epigenetic modifications refer mainly to DNA methylation and covalent modifications of the histone proteins. The tri-methylation of histone H3 lysine 4 (H3K4me3) and H3K36me3 have been associated with transcriptional activation, and H3K9me2 and H3K27me3 with gene silencing.^4^^,^^5^

H3K27me3 modification is catalyzed by the POLYCOMB REPRESSIVE COMPLEX 2 (PRC2), which is composed of a subset of the polycomb group (PcG) proteins.^6^ In *Arabidopsis thaliana*, H3K27me3 sites occur in euchromatin and not in transposable elements (TEs) or heterochromatin.^7^^,^^8^ In plants, H3K27me3 regions are usually limited to single genes, rarely extending into adjacent genes.^8–10^ Conservation of H3K27me3 sites between lines has been observed in *A. thaliana*, rice, and maize.^9–11^ The chromosomal distribution of the H3K27me3 sites in the histones of genomes of different plant species provides data on the possible conservation of H3K27me3 sites and effect on gene activity.^6^ H3K27me3 preferentially marks repressed or lowly expressed genes in *A. thaliana*, rice, and maize.^12^^,^^13^ The level of H3K27me3 can vary between tissues at the same gene locus.^8^^,^^10^^,^^14–16^

In *A. thaliana*, *FLOWERING LOCUS C* (*FLC*) is a key determinant of vernalization, which is the acquisition or acceleration of the ability to flower by a prolonged low temperature treatment.^17^*FLC* encodes a MADS box DNA-binding protein, which acts as a floral repressor of *SUPPRESSOR OF CONSTANS OVEREXPRESSION 1* (*SOC1*) and *FLOWERING LOCUS T* (*FT*).^18–20^ Without prolonged cold exposure, *FLC* is expressed. With cold exposure chromatin structure at *FLC* is remodelled from an active to a repressed state. H3K27me3 levels are increased following vernalization, this is catalyzed by the VERNALIZATION (VRN) complex, one of several PRC2 complexes, and *FLC* expression is reduced.^21^^,^^22^ The FLC protein belongs to a MADS-box protein family, which contains five other members, MADS AFFECTING FLOWERING 1–5 (MAF1–MAF5). *MAF1*–*MAF4* are repressed by vernalization, and the repression in each case is associated with an increase of H3K27me3 level.^23^

The control of flowering is a critical property for the Chinese cabbage crop.^24^ Chinese cabbage is generally vernalization sensitive and can respond to cold exposure during seed germination.^25–27^ In the absence of vernalization, most Chinese cabbage lines do not bolt before 6 months after sowing.^27^ There are four *FLC* paralogs (*BrFLC1*, *BrFLC2*, *BrFLC3*, and *BrFLC5*) in the genome,^28^ and all can be repressed by vernalization treatment in some Chinese cabbage lines.^29–32^

In the present study, two inbred lines of Chinese cabbage, RJKB-T23 (T23) and RJKB-T24 (T24) show high sequence similarity to the reference genome of *B. rapa* var. *pekinensis* Chiifu-401-42 version 1.5,^28^ have similar genetic distances to the reference genome,^33^ and are parents of the commercial F_1_ hybrid cultivar, ‘W77’. We compared the distribution of H3K27me3 in the cotyledons and earliest leaves of these parental inbred lines to investigate the role of H3K27me3 in tissue-specific gene expression. We found more similarity of distribution between the two parental lines than between different tissues within a line. In paralogous gene families and 14-day tissue, the presence of H3K27me3 was associated with tissue-specific gene expression. We showed that H3K27me3 plays an important role in the regulation of *FLC* paralogous genes in the vernalization process of *B. rapa*.

## 2. Materials and methods

### 2.1. Plant materials and growth conditions

Two Chinese cabbage inbred lines (*B. rapa* var. *pekinensis*), RJKB-T23 (T23)/R09 and RJKB-T24 (T24)/S11,^34^^,^^35^ and the C24 accession of *A. thaliana* were used. The genetic relationship between RJKB-T23 and RJKB-T24 is shown in Supplementary Fig. S1*. B. rapa* seeds were surface sterilized and grown on agar-solidified Murashige and Skoog (MS) plates with 1% (w/v) sucrose under long day (LD) condition (16 h light) at 22 °C. Plants were harvested at 2 and 14 days after sowing [2 days; cotyledons (2d-C), 14 days; 1st and 2nd leaves (14d-L)] for expression and ChIP analyses (Supplementary Fig. S2). *A. thaliana* seeds were surface sterilized and grown on MS with 3% (w/v) sucrose agar medium. After a 2-day stratification period at 4 °C, seedlings were grown at 22 °C under LD condition (16 h light) for 12 days.

T23 and T24 respond to cold exposure (below 10 °C) and are both seed vernalization-responsive type. They do not flower within 4 months of sowing without vernalization. For vernalizing cold treatments in T24, seeds were surface sterilized and placed on agar-solidified MS plates with 1% (w/v) sucrose for 4 weeks at 4 °C under LD condition (16 h light). Plant materials were harvested at the end of 4 weeks cold treatment (cotyledons/BrV1) at which time the developmental stage was similar to seedlings at 2 days after sowing under normal growth conditions (2d-C). After 4 weeks of vernalization, seedlings were grown for 12 days using normal growth condition (1st and 2nd leaves/BrV2) at which time the developmental stage was similar to seedlings at 14 days after sowing under normal growth conditions (14d-L).

The C24 accession of *A. thaliana* is a vernalization-sensitive and seed vernalization-responsive type.^36^ For vernalization, C24 seeds were exposed to 4 °C for 4 weeks then grown at 22 °C under LD for 10 days (V) at which time the developmental stage was similar to seedlings at 12 days after sowing under normal growth conditions (NV).

### 2.2. Chromatin immunoprecipitation sequencing

Chromatin immunoprecipitation sequencing (ChIP-seq) experiments were performed as described by Buzas et al.^37^ One gram of cotyledons or 1st and 2nd leaves of *B. rapa* or seedlings of *A. thaliana* was used for ChIP analysis, and anti-H3K27me3 (Millipore, 07-449) antibodies were used. In *B. rapa*, before the ChIP-seq, we validated the enrichment of purified immunoprecipitated DNAs by qPCR using the positive and negative control primer sets of H3K27me3 previously developed (Supplementary Fig. S3, Table S1).^31^ Purified immunoprecipitated DNA and input DNA in *B. rapa* were sequenced by Hiseq2000 (36 bp single end). ChIP DNA fragments and input DNA in *A. thaliana* were sequenced by Illumina with an Illumina Genome Analyzer (GAII). Low quality reads or adapter sequences were purged from the ChIP-seq reads using cutadapt version 1.7.1 and Trim Galore! version 0.3.7. The reads were mapped to the *B. rapa* reference genome v.1.5 (http://brassicadb.org/brad/) or to the *A. thaliana* reference genome (TAIR 10) using Bowtie2 version 2.2.3 (Supplementary Tables S2 and S3). The two replicates of 14-day 1st and 2nd leaves in *B. rapa* showed high correlation (Supplementary Fig. S4), thus these two data sets were combined. The mapped reads on the interspersed repeat regions (IRRs), such as the TEs detected by RepeatMasker, were examined.

ChIP-qPCR was performed using a LightCycler Nano (Roche). The immunoprecipitated DNA was amplified using FastStart Essential DNA Green Master (Roche). PCR conditions were 95 °C for 10 min followed by 40 cycles of 95 °C for 10 s, 60 °C for 10 s, and 72 °C for 15 s, and melting program (60 °C to 95 °C at 0.1 °C/s). After amplification cycles, each reaction was subjected to melt temperature analysis to confirm single amplified products. Data presented are the average and standard error (SE) from three biological and experimental replications. Enrichment of H3K27me3 marks was calculated by comparing the target gene and non-H3K27me3-marked genes by qPCR using immunoprecipitated DNA as a template. The difference between primer pairs was corrected by calculating the difference observed by qPCR amplifying the input DNA as a template. Primer sequences used in this study are shown in Supplementary Table S1.

### 2.3. Detection of H3K27me3 peaks by model-based analysis for ChIP-seq

We performed peak calling on alignment results using model-based analysis for ChIP-seq (MACS) 2 2.1.0 and identified the regions having H3K27me3 peaks. The MACS callpeak was used with the following options: effective genome size: 2.30e + 08, band width: 200, model fold: 10–30, tag size: 36. The cut-off of *P*-value, 1.00e-05, was used to call significant peaks. H3K27me3-marked genes were defined as genes that had a more than 200-bp length peak within a genic region (exon–intron) including 200 bp upstream and downstream. The number of H3K27me3 peaks in *B. rapa* and *A. thaliana* is shown in Supplementary Tables S4 and S5, respectively. We validated H3K27me3-marked genes by ChIP-qPCR of genes that have been identified previously (Supplementary Fig. S5).^31^

### 2.4. Identification of differentially marked H3K27me3 genes

To statistically identify the difference of H3K27me3 genic regions between lines, tissues, or between non-vernalized and vernalized samples, mapped reads on a target region that contains a gene, 200 bp upstream and 200 bp downstream were counted. The number of ChIP-seq reads mapped on a target region was then normalized with signal extraction scaling (SES) method.^38^ Statistical significance of differences between samples was determined by Fisher’s exact test. The regions that showed significant differences of H3K27me3 level were selected with more than |log 2| = 2.0, *q*-value < 0.05 and, with significant H3K27me3 peaks.

### 2.5. Calculation of tissue specificity by *T*-value

We used RNA sequencing (RNA-seq) datasets that contain root, stem, leaf, flower, silique and callus samples.^39^ The tissue specificity index *T* (*T*-value) was computed in each gene according to the formula as described in Tong et al.^39^

### 2.6. Identification of SNPs in *B. rapa* genes

We performed whole genome resequences in T23 and T24.^33^ The reads of two lines were mapped to the *B. rapa* reference genome v.1.5 using Bowtie2 version 2.2.3. We used Picard v.2.9.0 ‘MarkDuplicates’ command to remove duplication. Samtools v1.7 ‘mpileup’ command with -q 20 -Q 30 options and bcftools v1.7 ‘call’ command with -p 0.9 -v -c -O z options were performed to make VCF files. The identification of SNPs was performed using bcftools ‘view’ command with -v snps -g hom options. SNPs that have more than 10 reads were used for subsequent analyses. The number of SNPs per nucleotide length was measured for each gene.

### 2.7. Gene ontology analysis

Analysis for enrichment of gene functional ontology terms was completed using the gene ontology (GO) tool agriGO^40^ following the methods described by Shimizu et al.^41^ Statistical tests for enrichment of functional terms used the hypergeometric test and false discovery rate (FDR) correction for multiple testing to a level of 1% FDR.

### 2.8. RNA extraction and qPCR

Total RNA was isolated from cotyledons (2d-C, BrV1) or 1st and 2nd leaves (14d-L, BrV2) using the SV Total RNA Isolation System (Promega). cDNA was synthesized from 500 ng total RNA using ReverTra Ace qPCR RT Master Mix with gDNA Remover (Toyobo). qPCR was performed by the same methods as the ChIP-qPCR using the cDNA as a template. The relative expression level of each gene relative to *ACTIN*^42^ was automatically calculated using automatic CQ calling according to the manufacturer’s instructions (Roche). Data presented are the average and SE from three biological and experimental replicates. Primer sequences used in this study are shown in Supplementary Table S1.

In BrV1 and BrV2, the region covering part of the *BrFLC* gene was amplified using primers, which can amplify all *BrFLC* paralogs (*BrFLC1*, *BrFLC2*, *BrFLC3*, *BrFLC5*), using cDNA as templates. PCR was performed using the following conditions: 1 cycle of 94 °C for 2 min, 35 cycles of 94 °C for 30 s, 58 °C for 30 s, and 68 °C for 30 s. Primer sequences used for RT-PCR are shown in Supplementary Table S1.

### 2.9. RNA sequencing

RNA-seq using 2-day cotyledons and 14-day leaves with two replicates was performed in T23 and T24 (100 nt read length with paired end on an Illumina HiSeq^TM^ 2000) conditions. The number of clean reads and the percentage of mapped reads are shown in Supplementary Table S6. Low quality reads were filtered using FaQCs Version 1.34.^43^ HISAT2^44^ was used to align the sequence data sets to Brapa_sequence_v1.5 (http://brassicadb.org/brad/). Cuffdiff was used for the gene expression levels scored by fragments per kilo-base per million (FPKM) and identification of differentially expressed genes (DEGs) with two criteria, 2-fold difference (log 2 ratio ≥ 1.0) and 95% confidence. The level of gene expression was categorized into seven groups using log 2 score of FPKM, e.g. Group-6 (highest), log 2 score of FPKM (x) is greater than 9.0; Group-5, 6.0<=x < 9.0; Group-4, 3.0<=x < 6.0; Group-3, 0.0<=x < 3.0; Group-2, -3.0<=x < 0.0; Group-1, x<-3.0; Group-0 (lowest), no read.^31^^,^^45^

## 3. Results

### 3.1. The two parental lines of *B. rapa* have conserved sites of H3K27me3 but H3K27me3 differs between tissues

**Figure 1:**
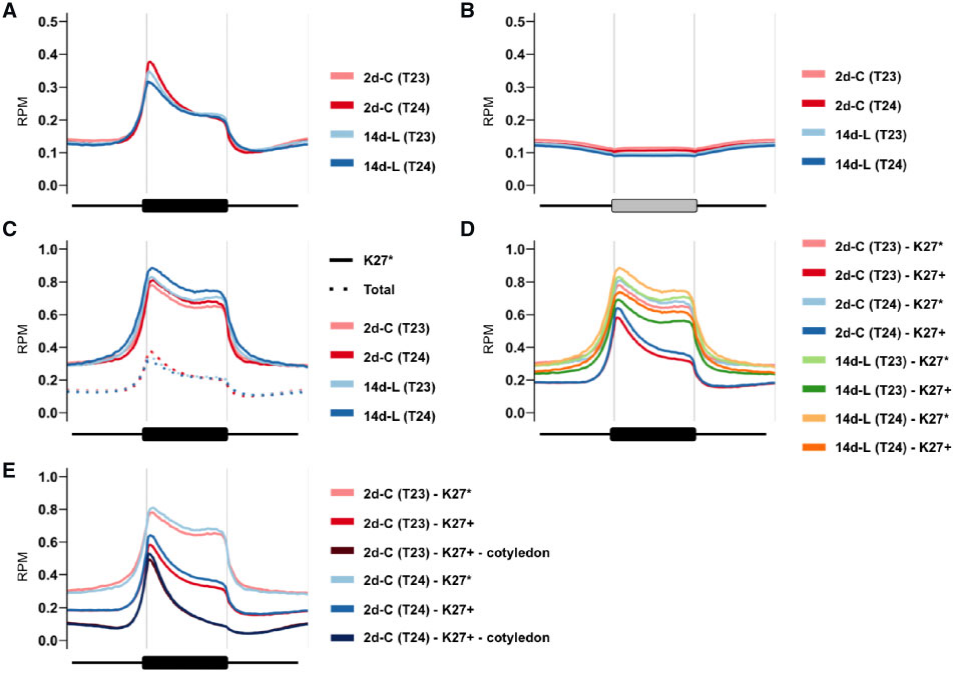
Metagene plots of H3K27me3 in genic region and IRR. (A, B) H3K27me3 level at genic or IRR region with 1 kb upstream and 1 kb downstream is shown using all genes (A) or all IRRs (B) in 2-day cotyledons (2d-C) and 14-day leaves (14d-L) in RJKB-T23 (T23) and RJKB-T24 (T24). (C, D) H3K27me3 level at genic region in all H3K27me3-marked genes (Total), H3K27me3 stably marked genes (K27*), or H3K27me3-marked genes (K27+) in 2d-C and 14d-L of both lines. (E) H3K27me3 level at genic region in H3K27me3 stably marked genes (K27*), H3K27me3-marked genes (K27+), or 2-day cotyledon-specific H3K27me3-marked genes (K27+ - cotyledon) in 2d-C of both lines. RPM: reads per million mapped reads.

The diversity and conservation of H3K27me3 distribution between parental lines of the commercial F_1_ hybrid cultivar of Chinese cabbage (T23 and T24) were determined. The presence of H3K27me3 marks on the chromatin of 2-day cotyledons (2d-C) and 14-day leaves (14d-L) in the parental lines was mapped by ChIP-seq (Supplementary Table S2). Reads mapped in the genic regions were classified into 2 kb upstream, exon, intron, and 2 kb downstream segments. The proportions of reads in each of these segments were similar to those in the input DNA (Supplementary Fig. S6, Table S7), suggesting there is no preferential location of H3K27me3 in any of the coding or regulating regions. H3K27me3 was enriched in the transcribed region in both 2-day cotyledon and 14-day leaf samples in both lines, especially around the transcription start sites (Fig. 1A). The percentage of reads in the IRRs (TEs and repeats) was lower than in the input DNA (Supplementary Table S2). There was no enrichment in IRR sequences or their flanking regions (Fig. 1B).

**Figure 2:**
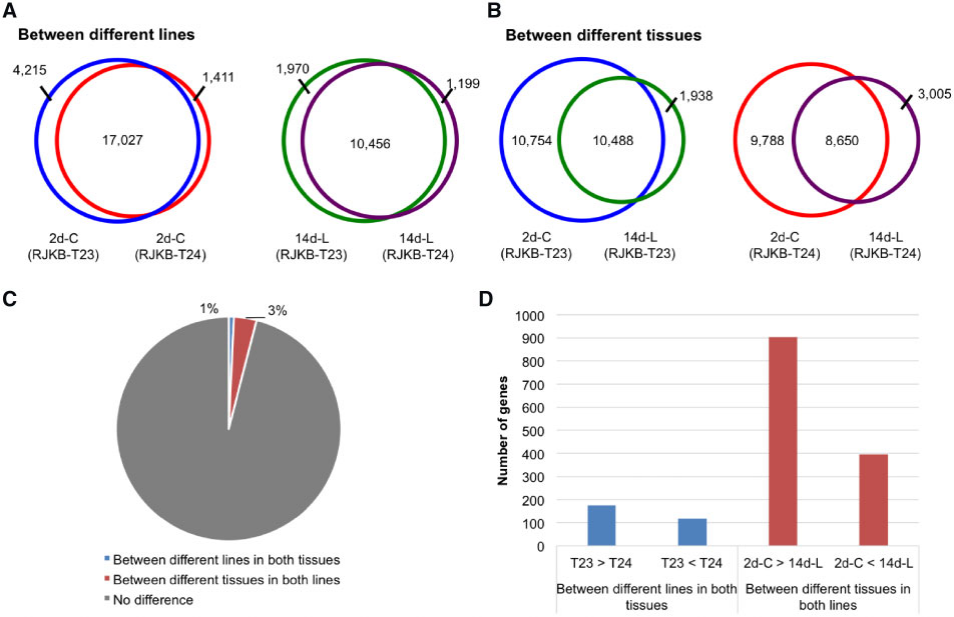
Comparison of H3K27me3 between lines or between tissues. (A, B) Venn diagram of genes having H3K27me3 in genic regions of 2-day cotyledons (2d-C) and 14-day leaves (14d-L) in RJKB-T23 (T23) compared with RJKB-T24 (T24). (C) Percentage of genes showing different H3K27me3 levels between lines in both tissues or between tissues in both lines. (D) Number of genes showing different H3K27me3-marked genes between lines in both tissues or between tissues in both lines.

We found a similar pattern of H3K27me3 distribution in the parental lines in two different tissues (2-day cotyledons and 14-day leaves) at the whole genome level (Supplementary Fig. S7A). We defined an H3K27me3-marked gene as having a peak of more than 200 bp within the genic region, which includes 200 bp upstream and downstream sequences (see Section 2). A total of 17,027 H3K27me3-marked genes were common to the two parental lines in the 2-day cotyledons, and 10,456 H3K27me3-marked genes in the 14-day leaf samples were present in both lines (Fig. 2A, Supplementary Table S8). The 2-day cotyledon sample contained most of the marked 14-day genes as well as 2-day cotyledon specific sites (Fig. 2B). A total of 7,930 H3K27me3-marked genes were present in 2-day cotyledon and 14-day leaf samples in both lines and were termed H3K27me3 stably marked genes. In these genes, H3K27me3 was enriched throughout the transcribed region (Fig. 1C).

We compared H3K27me3 levels by the number of ChIP-seq reads that had been normalized using SES (see Section 2).^38^ The two parental lines showed few differences in H3K27me3 distribution in the same tissue contrasting to the differences between 2-day cotyledons and 14-day leaves (Fig. 2C and D, Supplementary Table S9). In genic regions, the correlation coefficient between tissues was lower than between lines (Supplementary Fig. S7B).

### 3.2. At 14 days but not 2 days, genes marked with H3K27me3 had lower levels of transcription

There was a higher number of H3K27me3-marked genes in 2-day cotyledons compared with 14-day leaves (Supplementary Table S8). We examined whether these differences affected the transcriptome. We assigned genes into seven groups on the basis of their expression level in 14-day leaves of the two lines.^31^^,^^41^^,^^45^ Using the same criteria, we also categorized gene expression levels from a transcriptome of 2-day cotyledon material in each of the two lines (Supplementary Fig. S8).

**Figure 3:**
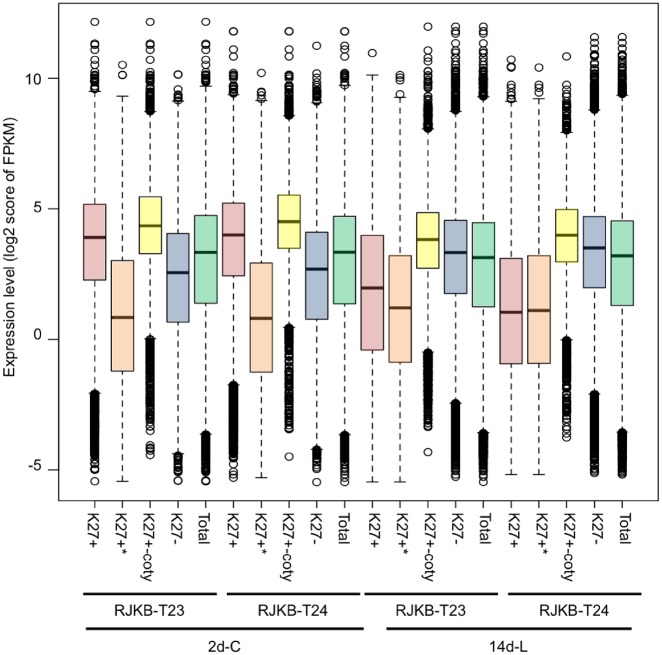
Box plots of the gene expression levels of log 2 score of FPKM with (+) or without (−) H3K27me3 in genic regions of RJKB-T23 and RJKB-T24. * indicates the log 2 score of FPKM in H3K27me3 stably marked genes. ‘Total’ indicates the log 2 score of FPKM in all genes (FPKM < 0.01). ‘K27+-coty’ represents the log 2 score of FPKM in 2-day cotyledon-specific H3K27me3-marked genes. FPKM: fragments per kilobase of transcript per million mapped reads, 2d-C: 2-day cotyledons, 14d-L: 14-day leaves.

In both the 2-day cotyledon and 14-day leaf samples in both lines, the proportion of H3K27me3-marked genes classified into each of the seven activity groups differs significantly from the distribution in the total transcriptome (Chi-squared test, *P *<* *10^−10^) (Supplementary Fig. S8). In the 2-day cotyledon samples, H3K27me3-marked genes were less frequently classified in Group-0 to -3 (low expression levels) and more frequently in Group-4 to -6 (high expression levels) (Supplementary Fig. S8). The average transcription level of H3K27me3-marked genes was higher than that of genes without H3K27me3 or in all of the genes in the two lines (Fig. 3). In contrast, in 14-day leaf material H3K27me3-marked genes had decreased numbers in Group-4 to -6 and increased numbers in Group-0 to -3 (Supplementary Fig. S8). The average transcription level of H3K27me3-marked genes was lower than that of genes without H3K27me3 or the total genes in both lines (Fig. 3). The average transcription level of H3K27me3 stably marked genes was lower than that of genes without H3K27me3 or than total genes in both tissues and lines (Fig. 3); the lower expression level could be due to the highly enriched H3K27me3 mark throughout gene body regions (Fig. 1C).

Contrary to expectation, H3K27me3-marked genes in 2-day cotyledons showed higher gene expression than those without marks (Fig. 3). These genes included 9,700 genes that had H3K27me3 only in 2-day cotyledons. The overall level of H3K27me3 was lower in the 2-day sample than in the 14-day sample, and H3K27me3 marks were significantly lower in the 3′ regions (Fig. 1D). Genes having H3K27me3 marks only in 2-day cotyledons were further decreased in the H3K27me3 level in the 3′ regions (Fig. 1E). The expected negative correlation between H3K27me3-marked genes and reduced expression was masked in the 2-day data because there are both highly expressed genes with a low level of H3K27me3 and lowly expressed genes with a higher level of H3K27me3.

### 3.3. H3K27me3 is associated with tissue-specific gene expression

In genes having different levels of H3K27me3 in the parental lines, 33.1% (241 of 729 genes in 2-day cotyledons) and 22.3% (200 of 898 genes in 14-day leaves) of genes showed differential expression levels (Supplementary Table S10). From 7.8% to 12.7% of these genes showed a negative correlation between H3K27me3 levels and expression levels between lines (Supplementary Table S10). There was a high proportion of genes with parallel levels of expression and of H3K27me3 (Supplementary Table S10), suggesting that the difference in expression between lines does not result from a difference in H3K27me3 level. In genes with different levels of H3K27me3 between tissues, approximately 60% of genes showed differential expression (Supplementary Table S10). More genes (from 13.3% to 19.8%) showed a negative correlation between a difference of H3K27me3 levels and expression levels between tissues, especially in genes showing higher H3K27me3 levels in 14-day leaves than in 2-day cotyledons (Supplementary Table S10).

**Figure 4:**
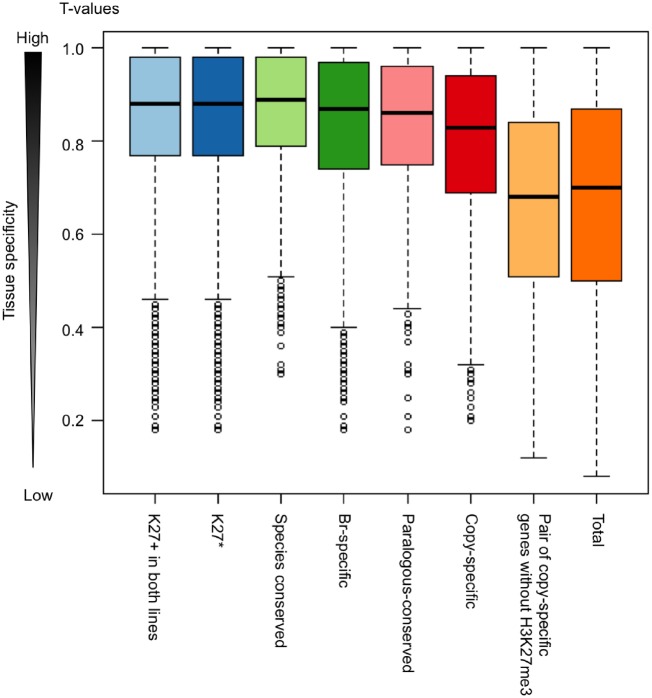
Tissue specificity of expression in genes having a H3K27me3 mark. A tissue specificity index, *T*-value, which interpolates the entire range between 0 for housekeeping genes and 1 for strictly one-tissue-specific genes, was calculated using the transcriptome data in six different tissues in *B. rapa.* ‘K27+’ and ‘K27*’ represent the H3K27me3-marked genes and H3K27me3 stably marked genes, respectively.

A tissue specificity index, *T*-value, which interpolates the entire range between 0 for housekeeping genes and 1 for strictly one tissue-specific genes, was calculated using the transcriptome data from six different tissues in *B. rapa*.^39^ We found H3K27me3-marked genes showed significantly higher average *T*-values compared with total genes (Fig. 4), suggesting that H3K27me3 has a role in tissue-specific gene expression.

### 3.4. Comparison of H3K27me3 states between paralogous genes in *B. rapa*

**Figure 5:**
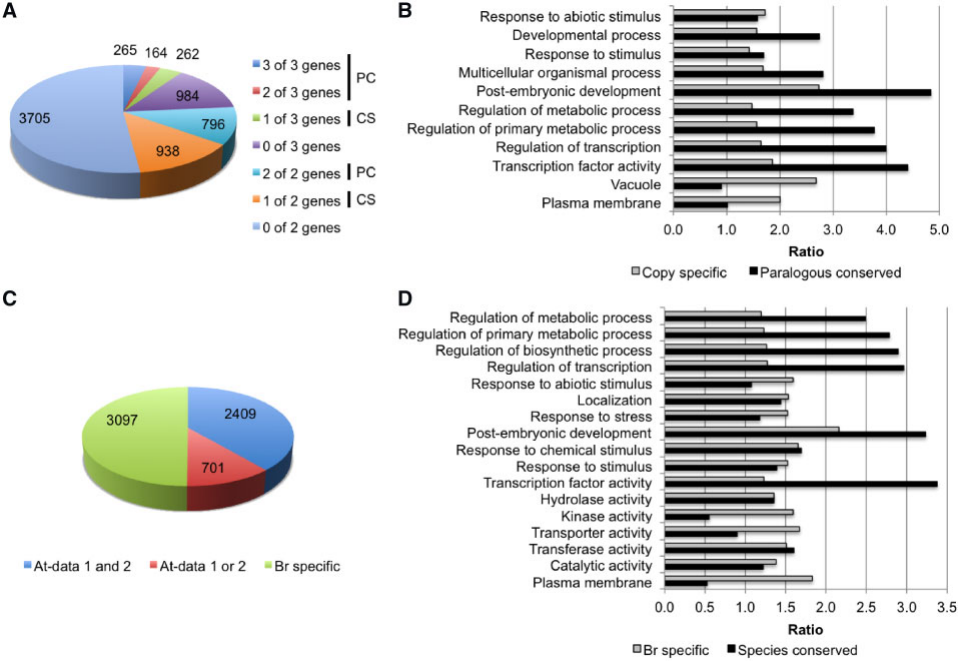
Analysis of paralogous and species-conserved genes. (A) Classification of paralogous conserved (PC) and copy-specific (CS) H3K27me3-marked genes. (B) GO analysis using paralogous-conserved and copy-specific H3K27me3-marked genes. (C) Classification of H3K27me3-marked genes in *B. rapa* (Br). Blue, species-conserved H3K27me3-marked genes; red, single data set of *A. thaliana* (At) genes that overlapped with *B. rapa*, green, *Br*-specific H3K27me3-marked genes. (D) GO analysis using species-conserved and *Br*-specific H3K27me3-marked genes.

We compared H3K27me3 locations between paralogs in 14-day leaf samples using 5,439 and 1,675 genes with two or three syntenic copies.^1^ Among the 1,675 three-copy sets, 265 had H3K27me3 in all three copies, 164 had H3K27me3 in at least two copies, 262 had H3K27me3 in at least one copy, and 984 sets did not have H3K27me3 in any copies (Fig. 5A). Among the 5,439 two-copy pairs, 796 pairs had H3K27me3 in both copies, 938 pairs had H3K27me3 in one copy, and 3,705 pairs did not have H3K27me3 in any copies (Fig. 5A). Totally, 1,225 pairs (= 265 + 164 + 796) showed conservation of H3K27me3 among paralogs, termed paralogous-conserved H3K27me3-marked genes, and 1,200 pairs (=262 + 938) showed H3K27me3 in one of the paralogs, termed copy-specific H3K27me3-marked genes (Fig. 5A).

GO analysis showed that genes categorized into ‘Transcription factor activity’, ‘Regulation of metabolic process’, and ‘Developmental process’ were overrepresented in paralogous-conserved H3K27me3-marked genes compared with copy-specific H3K27me3-marked genes (Fig. 5B, Supplementary Table S11). Genes categorized into ‘Plasma membrane’ and ‘Vacuole’ were overrepresented in copy-specific H3K27me3-marked genes compared with paralogous-conserved H3K27me3-marked genes (Fig. 5B, Supplementary Table S11).

We examined whether a difference in H3K27me3 states between paralogs was associated with a different level of gene activity. Between paralogous pairs, the average expression levels of genes with H3K27me3 marks was lower than those without H3K27me3 in 14-day leaves of both T23 and T24 (Supplementary Fig. S9), indicating that the presence of H3K27me3 results in a difference of gene expression level between paralogous pairs. There was no difference in *T*-values between paralogous-conserved and copy-specific H3K27me3-marked genes, and *T*-values were higher than that in total genes (Fig. 4). Between paralogous pairs, the average *T*-values of genes with H3K27me3 was significantly higher than that without H3K27me3 (Fig. 4, Supplementary Fig. S10), suggesting an association of H3K27me3 with tissue-specific gene expression differences between paralogous pairs.

### 3.5. Some genes marked with H3K27me3 are shared between *B. rapa* and *A. thaliana*

To gain information about conservation of H3K27me3 states beyond *B. rapa*, we compared the genes marked with H3K27me3 in *B. rapa* and *A. thaliana*. Of 10,456 genes, which had H3K27me3 in the 14-day leaf samples in both *B. rapa* lines (Fig. 2A), 9,769 (93.4%) had sequence homology to putatively orthologous genes in *A. thaliana*. This was reduced to 6,207 genes in *A. thaliana* following the removal of genes where two or three *B. rapa* genes showed sequence similarity to one *A. thaliana* gene. In *A. thaliana*, 5,333 genes identified as having H3K27me3 marks in 12-day seedlings in the C24 accession (At-data 1),^46^ and 5,118 genes (At-data 2), which were selected by Berke and Snel.^47^ About 80% of genes overlapped between the two data sets (At-data 1 and At-data 2). Between orthologous genes of *B. rapa* and *A. thaliana*, 2,409 of 6,207 genes (38.8%) had H3K27me3 in both *B. rapa* and the two data sets of *A. thaliana*, these were termed species-conserved H3K27me3-marked genes. A total of 3,097 of 6,207 genes (49.9%) had H3K27me3 in only *B. rapa*, these were termed *Br*-specific H3K27me3-marked genes (Fig. 5C).

Genes categorized into ‘Transcription factor activity’ and ‘Regulation of metabolic process’ tended to be overrepresented in species-conserved H3K27me3-marked genes compared with *Br*-specific H3K27me3-marked genes (Fig. 5D, Supplementary Table S12). In contrast, genes categorized into ‘Plasma membrane’, ‘Transporter activity’, ‘Kinase activity’, ‘Response to stress’, and ‘Response to abiotic stimulus’ tended to be overrepresented in *Br*-specific H3K27me3-marked genes compared with species-conserved H3K27me3-marked genes (Fig. 5D, Supplementary Table S12).

Both species-conserved and *Br*-specific H3K27me3-marked genes showed high tissue specificity (Fig. 4). Genes having H3K27me3 tended to have a lower SNP number per length in each gene (mutation rates) than total genes as did species-conserved H3K27me3-marked genes relative to *Br*-specific H3K27me3-marked genes in T23 but not in T24 (Supplementary Fig. S11).

### 3.6. Higher H3K27me3 levels in 14-day leaves are associated with decreased post-embryonic gene expression

H3K27me3 plays an important role in tissue-specific gene expression. We identified genes showing different H3K27me3 states just after germination (2-day cotyledons) and more than 10 days after germination (14-day leaves) in *B. rapa* (Supplementary Table S9). We compared the H3K27me3 levels between germinating seeds and 12-day seedlings (At-data 1) in C24 accession of *A. thaliana* (Supplementary Table S13).^46^^,^^48^

Four genes showed higher H3K27me3 levels in 2-day cotyledons than in 14-day leaves in both *B. rapa* lines and in *A. thaliana,* in germinating seeds than in 12-day seedlings (Supplementary Table S13). Sixty-four genes showed higher H3K27me3 levels in 14-day leaves than in 2-day cotyledons in both *B. rapa* lines and in *A. thaliana*, in 12-day seedlings than in germinating seeds (Supplementary Table S13), and these genes tended to be categorized into ‘Seed maturation’, ‘Seed dormancy’, ‘Seed development’, and ‘Post-embryonic development’ (Supplementary Table S14). Genes categorized into ‘Post-embryonic development’ tended to have lower expression levels in 14-day leaves than in 2-day cotyledons in *B. rapa* (Supplementary Table S15), suggesting H3K27me3 is involved in silencing of embryogenic expression in 14-day leaves relative to their activity levels in 2-day cotyledons.

### 3.7. Change of H3K27me3 state after vernalization treatment in *B. rapa*

Vernalization involves regulation of gene expression of *FLC* by H3K27me3.^21^^,^^22^^,^^24^ We confirmed H3K27me3 accumulation at the *FLC* locus by a comparison of the whole genome level of H3K27me3 states between non-vernalized (NV) and upon return to 22 °C after vernalization (V) in the C24 accession of *A. thaliana*. In the genome, only *FLC* showed an increased H3K27me3 level following return to 22 °C after vernalization in *A. thaliana* (Supplementary Table S16).

**Figure 6:**
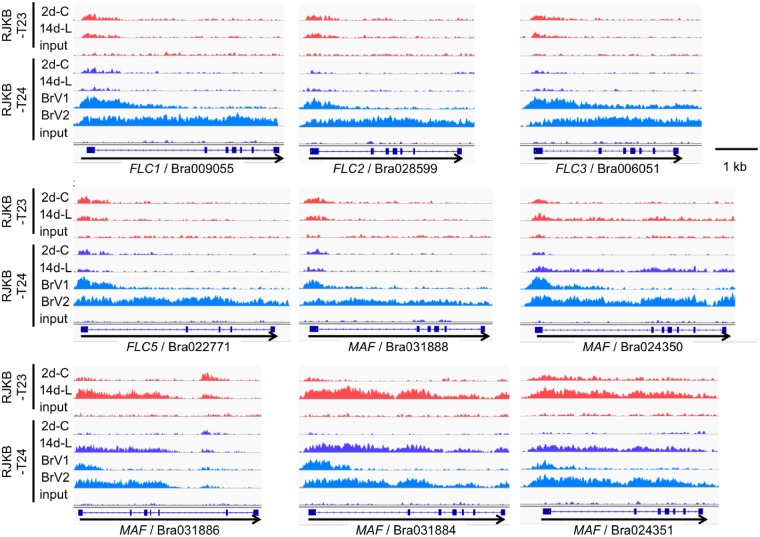
Visualization of H3K27me3 peaks by Integrative Genomics Viewer (IGV). 2d-C and 14d-L are non-vernalized samples and BrV1 and BrV2 are vernalized samples. 2d-C, 2-day cotyledons; 14d-L, 14-day leaves; BrV1, seeds were treated for 4 weeks at 4 °C (vernalization); BrV2, seeds were treated for 4 weeks at 4 °C and plants were transferred to the normal growth conditions for 12 days after vernalization.

We examined H3K27me3 states in non-vernalized (2d-C, 14d-L), vernalized (BrV1), and following return to 22 °C after vernalization (BrV2) in T24 (*B. rapa*)*.* Between non-vernalized and vernalized samples at similar developmental stages, H3K27me3 levels at the whole genome level had correlation coefficients of 0.97 and 0.99 (Supplementary Fig. S7C). Some genes showed increased H3K27me3 levels following vernalization including the four *BrFLC* paralogs (Supplementary Tables S16 and S17). Following 4 weeks vernalization (BrV1) H3K27me3 increased at the first exon and part of the first intron (nucleation site) in all four *FLC* paralogs (Fig. 6). Following a return to 22 °C after vernalization (BrV2), an increase of H3K27me3 was observed in all four *FLC* paralogs, which started at the nucleation site and spread 5′ to 3′ along the genes (Fig. 6). Expression levels of the total amount of the four *FLC* paralogs were downregulated in BrV1 and BrV2 compared with the unvernalized 2d-C and 14d-L, respectively (Supplementary Fig. S12). *Vernalization insensitive 3* (*VIN3*) and *SOC1* were highly induced in BrV1 and BrV2, respectively, and the expression level of *FT* was upregulated in BrV1 and BrV2 (Supplementary Fig. S12).

Three of five *MAF*-like genes (Bra031888, Bra024350, and Bra024351) showed a spread from 5′ to 3′ of H3K27me3 similar to that seen in the *FLC* genes following the return to normal conditions after 4 weeks of cold treatment (Fig. 6). Expression levels of these three *MAF*-like genes were downregulated following vernalization (Supplementary Fig. S12). Increased H3K27me3 levels of Bra006050 and Bra009056 in the sample upon return to 22 °C might be due to the spread of H3K27me3 from the nearby genes, Bra006051 (*BrFLC3*) and Bra009055 (*BrFLC1*) (Supplementary Fig. S13); other genes neighbouring *FLC* or *MAF* genes did not have increased H3K27me3 levels (Supplementary Fig. S14). From the comparison of ChIP-seq data, two genes (Bra032761/CYTOKININ-INDEPENDENT 1 and Bra037899/protein kinase family protein) showed decreased H3K27me3 levels following a return to 22 °C after vernalization in both *B. rapa* and *A. thaliana* (Supplementary Table S16).

## 4. Discussion

The location of H3K27me3 sites was conserved between the two *B. rapa* lines. The sites of H3K27me3 modification are less conserved between tissues than between lines, especially in genic regions. Genes with H3K27me3 marks have tissue-specific expression, suggesting a role for H3K27me3 in regulation of gene expression during development.

The H3K27me3-marked genes in 14-day leaves showed lower expression levels on average than total gene expression levels, but the H3K27me3-marked genes in 2-day cotyledons showed higher expression levels than the average level of gene expression. This was especially the case in 2-day cotyledon-specific H3K27me3-marked genes. Genes that are H3K27me3-marked in both 2-day cotyledons and 14-day leaves had lower expression levels on average than the total genes in both tissues. The level of H3K27me3 in 2-day cotyledon-specific H3K27me3-marked genes was lower around the 3′ region, while H3K27me3 stably marked genes showed a higher enrichment of H3K27me3 throughout the gene body region, suggesting that the low level of H3K27me3 present in 2-day cotyledon-specific H3K27me3-marked genes is not sufficient to repress gene expression. In general, there is an association between H3K27me3 marks on a gene and lower expression. In the 2-day cotyledons, this relationship did not hold. This finding is probably because of the early developmental time used; none of the analyses in other species used such early material.

Genes that showed higher levels of H3K27me3 in 14-day leaves compared with 2-day cotyledons showed an overrepresentation in the category of ‘Post-embryonic development’. These genes had lower expression levels in 14-day leaves than in 2-day cotyledons, suggesting a role in reducing the expression of genes concerned with embryo development at this time. In maize and *A. thaliana*, 34% of orthologues were marked.^10^ In maize and rice, 70% of orthologs are similarly marked.^10^ The data suggest that genes having important functions such as regulation of transcription factor gene expression or metabolic process also have conservation of H3K27me3 marks.

Approximately 30% of the pairs of paralogous genes in *B. rapa* had H3K27me3 in more than one copy. A similar situation holds with most pairs of syntenic genes in maize and *A. thaliana*.^10^^,^^49^ The tissue specificity of gene expression was higher in paralogous pairs marked with H3K27me3 than in those without H3K27me3 marks and expression levels were lower in the marked genes. These findings suggest that the different distribution of H3K27me3 between paralogous genes may be involved in their sub-functionalization.

Environmentally related changes at the *FLC* locus involve H3K27me3 marks. In *A. thaliana*, H3K27me3 has functional roles associated with the activity level of *FLC*.^21^^,^^22^^,^^24^ Plants treated for 4 weeks at 4 °C (vernalization) gain H3K27me3 at a site downstream of the transcription start site (the nucleation region), and gene expression ceases. Following return of the plants to 22 °C, H3K27me3 spreads across the entire *FLC* locus stabilizing the lack of expression, allowing expression of the floral promoters, *FT* and *SOC1*, and permitting flowering to occur.^50^ In *B. rapa*, *FLC* has a similar role in vernalization.^24^^,^^51^ Three *BrFLC* paralogs (*BrFLC1*, *BrFLC2*, and *BrFLC3*), which might have been generated by whole genome triplication (Supplementary Fig. S15), and *BrFLC5* had active transcription before vernalization. Their expression decreased after vernalization; the repressed state was maintained upon return to 22 °C after vernalization. Prior to the low temperature treatment in *B. rapa* all four *FLC* paralogs had only background levels of H3K27me3. At the end of 4 weeks low temperature treatment, H3K27me3 was localized at the proximal nucleation region. H3K27me3 spreads across all of the four *FLC* paralogs upon return to 22 °C after vernalization. In both *B. rapa* and *A. thaliana*, there is little change to genome-wide H3K27me3 levels following a return of the plants to 22 °C. There are five *MAF*-like genes in *B. rapa* and it is not clear whether these paralogs were generated by whole genome triplication (Supplementary Fig. S15). Spreading of H3K27me3 was also observed in some of the *MAF*-like genes.

In *A. thaliana*, a non-coding RNA COLDAIR molecule derived from the first intron of *FLC* is reported to play a role in the recruitment of PRC2 to *FLC*.^52^ There is no sequence showing similarity to COLDAIR in any of the first introns of the four *FLC* paralogs in *B. rapa*.^51^^,^^53^ In agreement with Li et al.,^32^ we did not find any transcript from the first intron of any of the four *FLC* paralogs in *B. rapa* in vernalized leaves.^54^ COLDAIR and any other non-coding RNAs may not be expressed from the first intron of *FLC* paralogs in *B. rapa.*^54^ A stabilization of repression by the spreading of H3K27me3 marks on return to normal growth condition has been attributed to COLDAIR RNA control but the absence of COLDAIR transcripts in *B. rapa* suggests a reconsideration of this view is needed.

## Supplementary Material

dsz021_Supplementary_Data
