# Supplementary material for: The histone modification H3 lysine 27 tri-methylation has conserved gene regulatory roles in the triplicated genome of Brassica rapa L
Source: DNA Res. 2019 Oct 17;26(5):433–43. doi: 10.1093/dnares/dsz021 (PMC6796510; doi:10.1093/dnares/dsz021)
Supplement: dsz021_Supplementary_Data [file dsz021_supplementary_data.zip › dsz021-Suppl_data/Supplementary_Figures.pptx]

## Slide 1
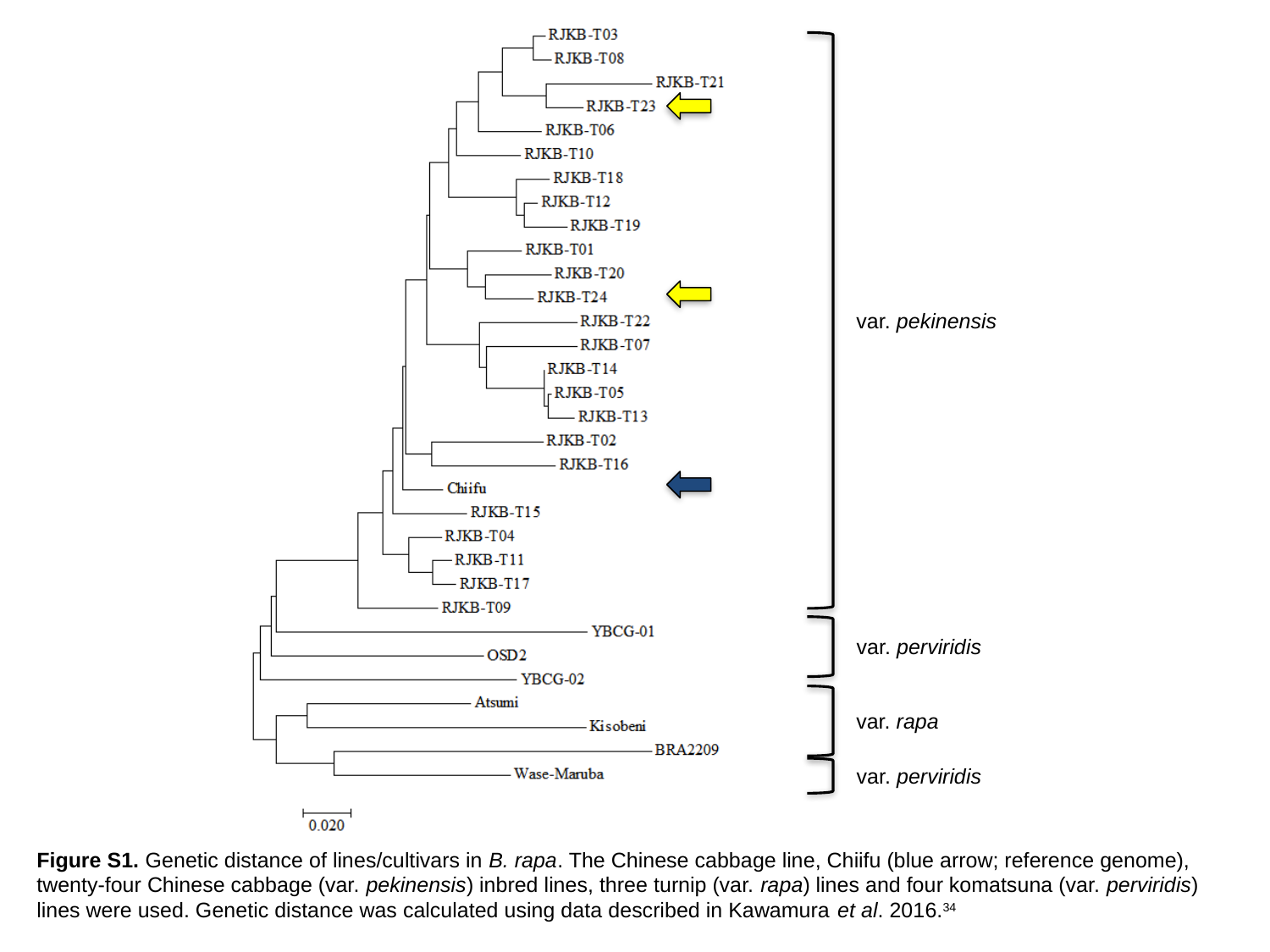

var. pekinensis
var. perviridis
var. rapa
var. perviridis
Figure S1. Genetic distance of lines/cultivars in B. rapa. The Chinese cabbage line, Chiifu (blue arrow; reference genome), twenty-four Chinese cabbage (var. pekinensis) inbred lines, three turnip (var. rapa) lines and four komatsuna (var. perviridis) lines were used. Genetic distance was calculated using data described in Kawamura et al. 2016.34

## Slide 2
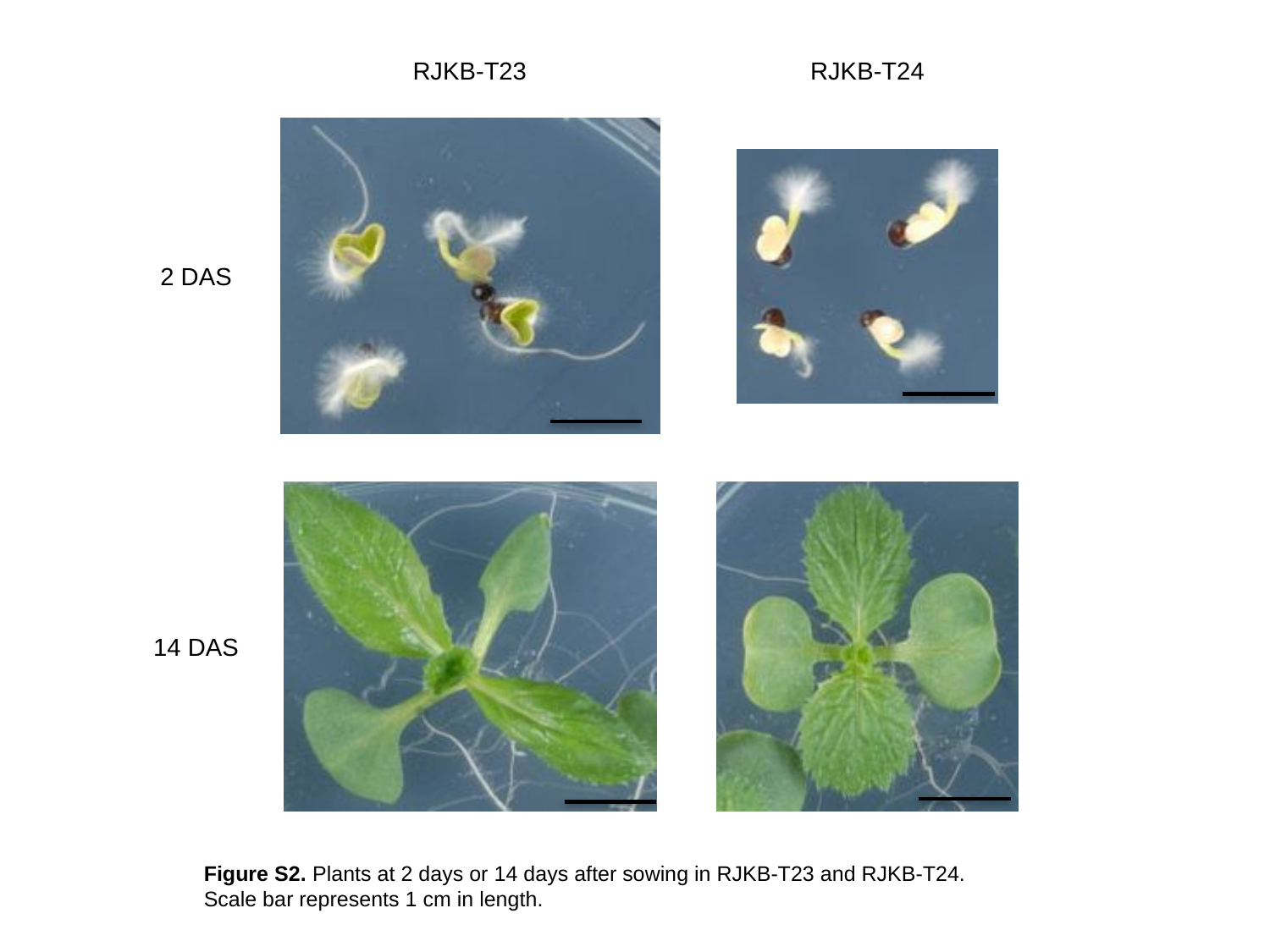

RJKB-T23
RJKB-T24
2 DAS
14 DAS
Figure S2. Plants at 2 days or 14 days after sowing in RJKB-T23 and RJKB-T24. Scale bar represents 1 cm in length.

## Slide 3
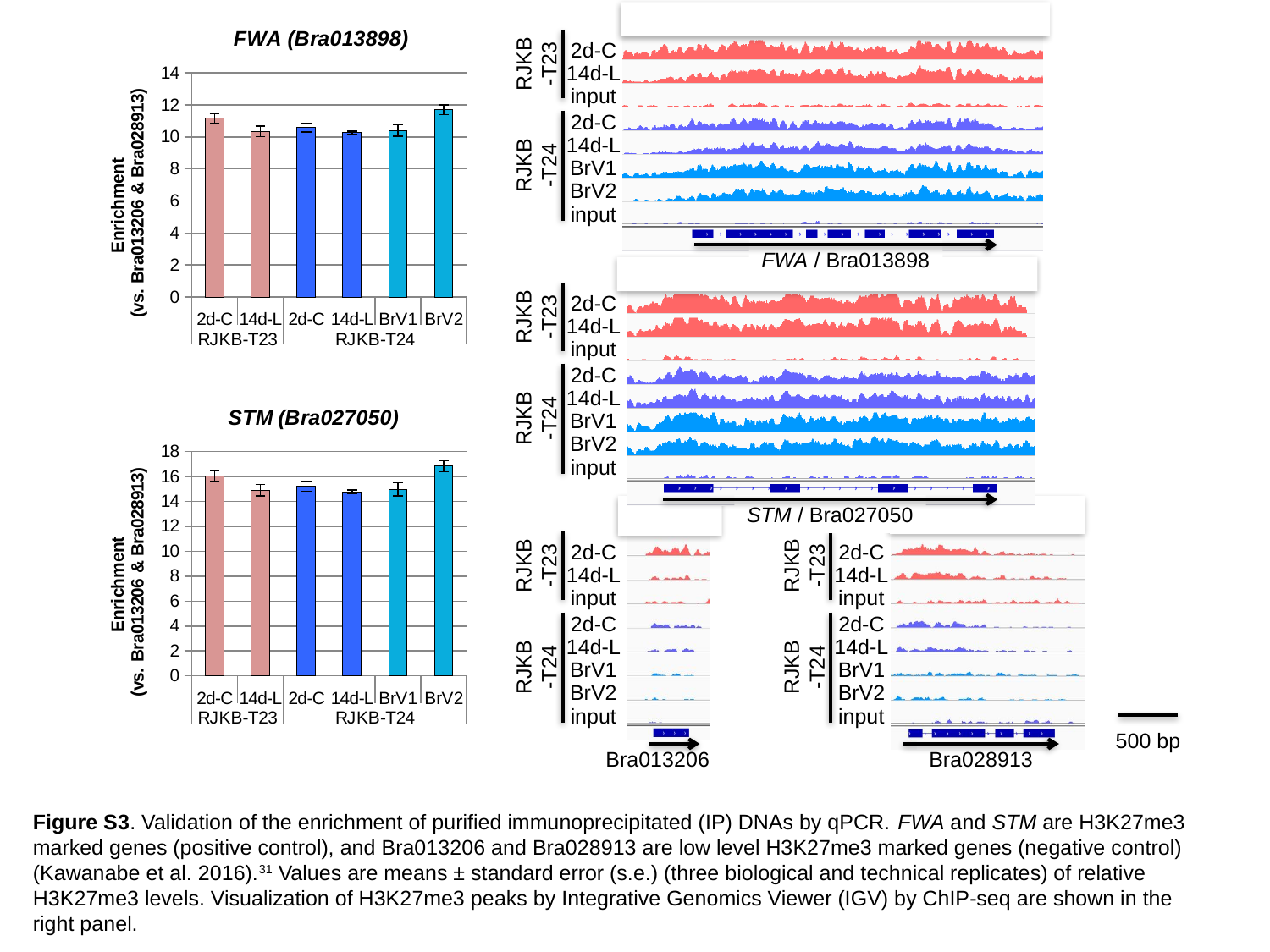

### Chart: FWA (Bra013898)
| Category | |
|---|---|
| 2d-C | 11.15045833365107 |
| 14d-L | 10.34779157399249 |
| 2d-C | 10.57996321203674 |
| 14d-L | 10.25987493195918 |
| BrV1 | 10.41016506488184 |
| BrV2 | 11.68746789855611 |
2d-C
RJKB
-T23
14d-L
input
2d-C
14d-L
RJKB
-T24
BrV1
BrV2
input
FWA / Bra013898
2d-C
RJKB
-T23
14d-L
input
2d-C
14d-L
RJKB
-T24
BrV1
BrV2
input
STM / Bra027050
### Chart: STM (Bra027050)
| Category | |
|---|---|
| 2d-C | 16.04382494050514 |
| 14d-L | 14.88890873955754 |
| 2d-C | 15.22296865041257 |
| 14d-L | 14.762409974042 |
| BrV1 | 14.97865476961416 |
| BrV2 | 16.81650057346202 |
2d-C
RJKB
-T23
14d-L
input
2d-C
14d-L
RJKB
-T24
BrV1
BrV2
input
Bra013206
2d-C
RJKB
-T23
14d-L
input
2d-C
14d-L
RJKB
-T24
BrV1
BrV2
input
Bra028913
500 bp
Figure S3. Validation of the enrichment of purified immunoprecipitated (IP) DNAs by qPCR. FWA and STM are H3K27me3 marked genes (positive control), and Bra013206 and Bra028913 are low level H3K27me3 marked genes (negative control) (Kawanabe et al. 2016).31 Values are means ± standard error (s.e.) (three biological and technical replicates) of relative H3K27me3 levels. Visualization of H3K27me3 peaks by Integrative Genomics Viewer (IGV) by ChIP-seq are shown in the right panel.

## Slide 4
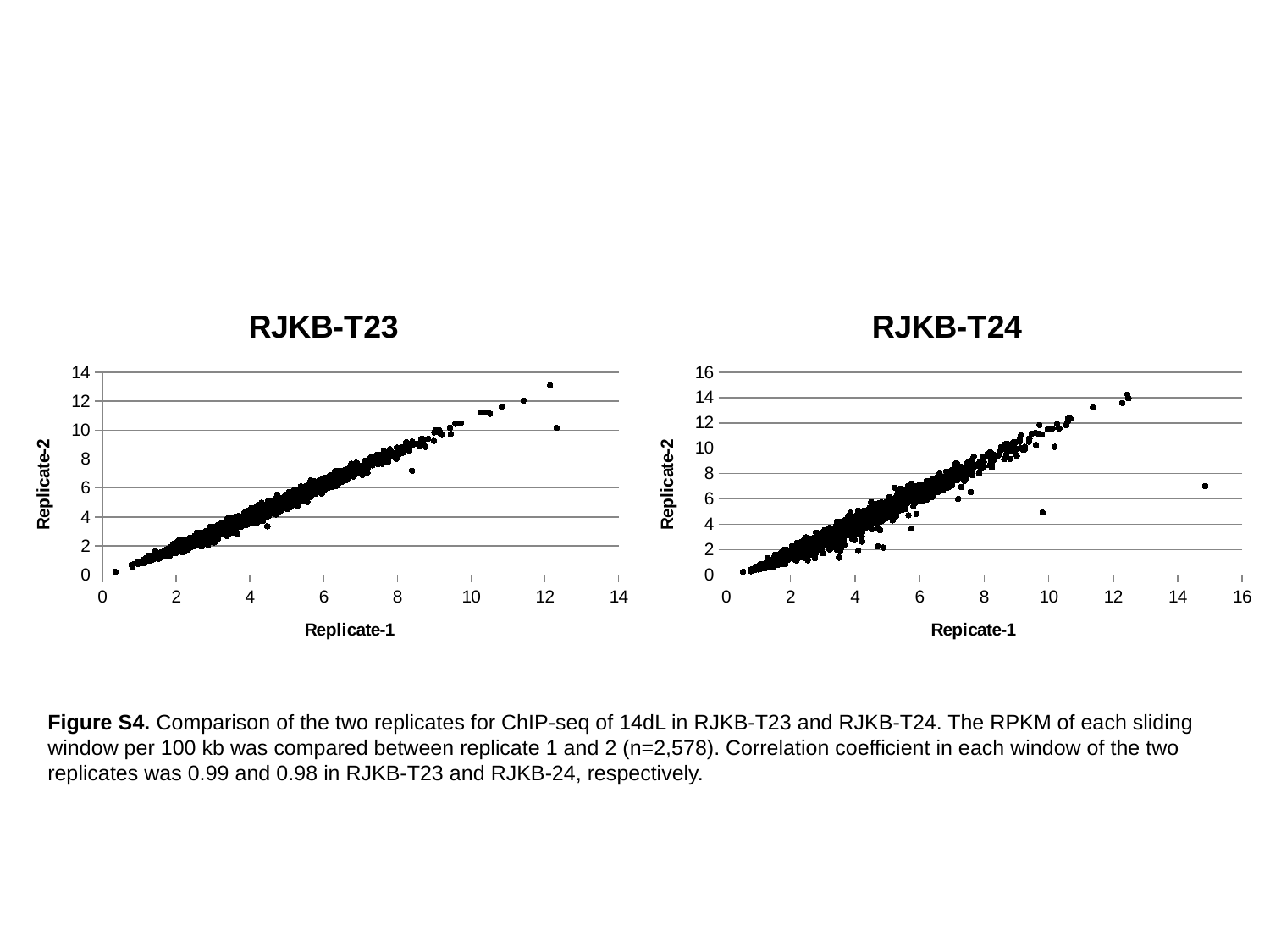

### Chart: RJKB-T23
| Category | ip.m.rep2 |
|---|---|
### Chart: RJKB-T24
| Category | ip.w.rep2 |
|---|---|Figure S4. Comparison of the two replicates for ChIP-seq of 14dL in RJKB-T23 and RJKB-T24. The RPKM of each sliding window per 100 kb was compared between replicate 1 and 2 (n=2,578). Correlation coefficient in each window of the two replicates was 0.99 and 0.98 in RJKB-T23 and RJKB-24, respectively.

## Slide 5
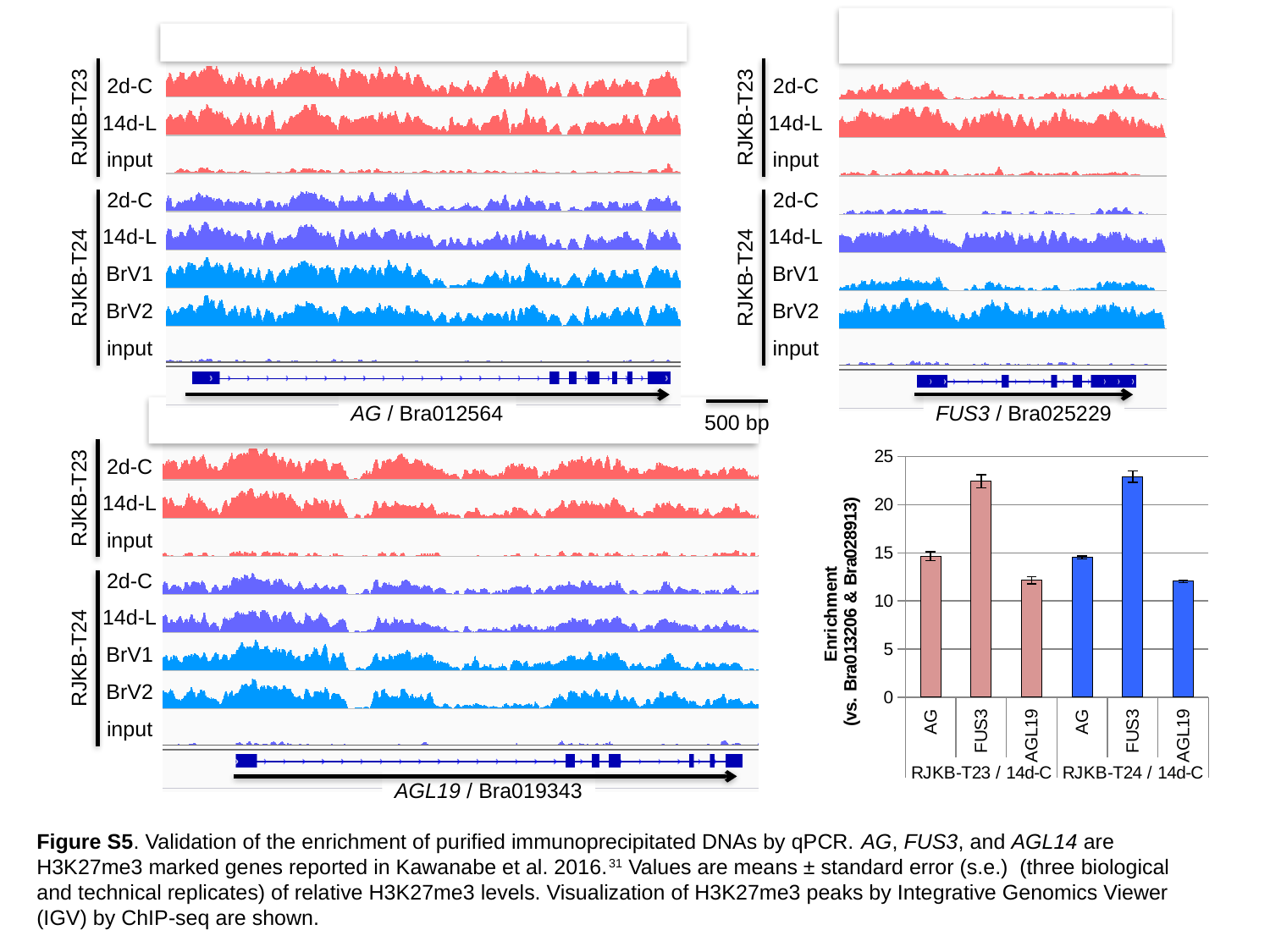

2d-C
RJKB-T23
14d-L
input
2d-C
14d-L
BrV1
RJKB-T24
BrV2
input
FUS3 / Bra025229
2d-C
RJKB-T23
14d-L
input
2d-C
14d-L
BrV1
RJKB-T24
BrV2
input
AG / Bra012564
2d-C
RJKB-T23
14d-L
input
2d-C
14d-L
BrV1
RJKB-T24
BrV2
input
AGL19 / Bra019343
500 bp
### Chart
| Category | |
|---|---|
| AG | 14.63330515604583 |
| FUS3 | 22.4292144531532 |
| AGL19 | 12.14117021680658 |
| AG | 14.50897804315715 |
| FUS3 | 22.93245491972022 |
| AGL19 | 12.03801671702995 |Figure S5. Validation of the enrichment of purified immunoprecipitated DNAs by qPCR. AG, FUS3, and AGL14 are H3K27me3 marked genes reported in Kawanabe et al. 2016.31 Values are means ± standard error (s.e.) (three biological and technical replicates) of relative H3K27me3 levels. Visualization of H3K27me3 peaks by Integrative Genomics Viewer (IGV) by ChIP-seq are shown.

## Slide 6
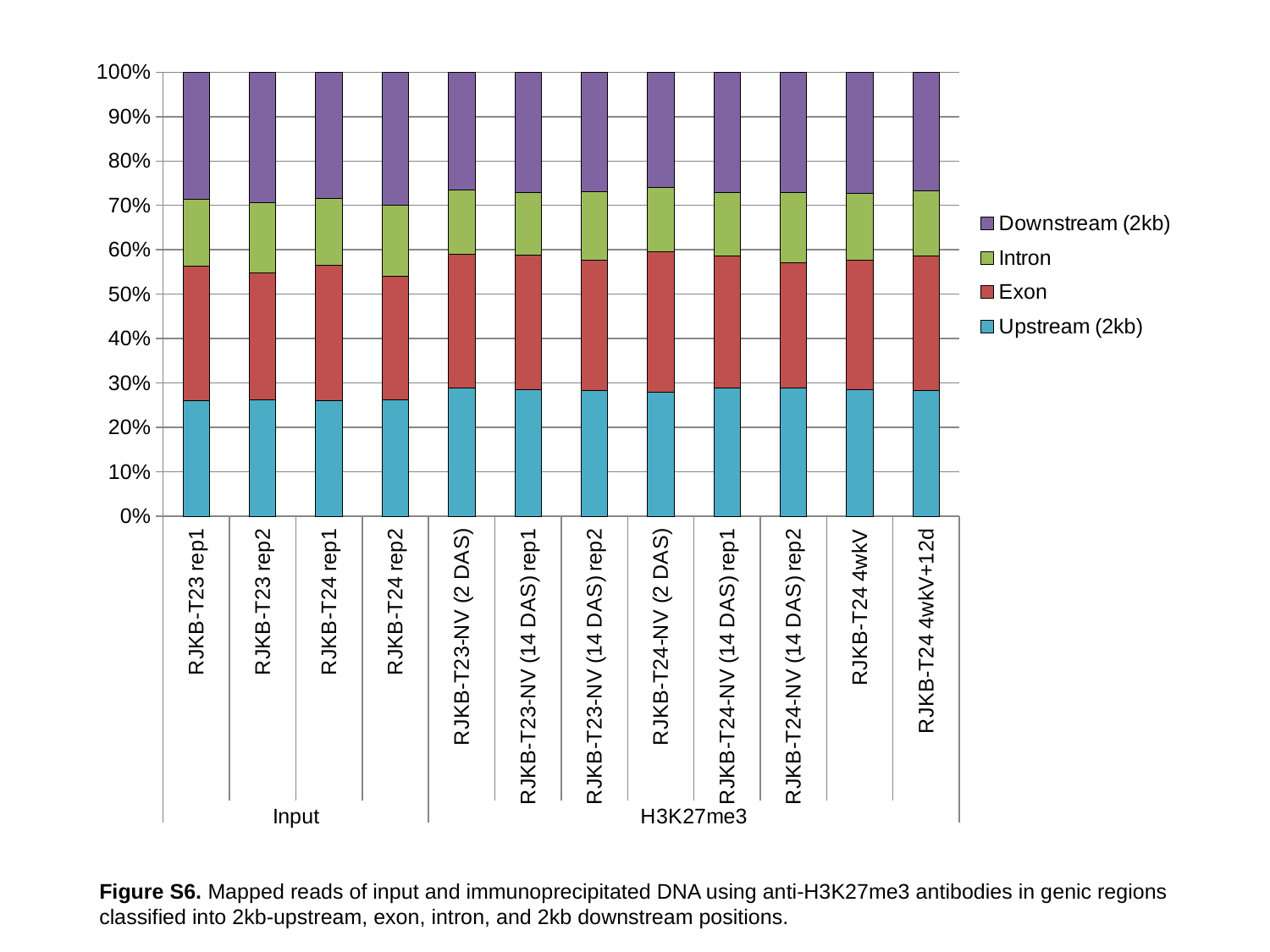

### Chart
| Category | Upstream (2kb) | Exon | Intron | Downstream (2kb) |
|---|---|---|---|---|
| RJKB-T23 rep1 | 3104668.0 | 3617693.0 | 1815451.0 | 3410205.0 |
| RJKB-T23 rep2 | 4185124.0 | 4588848.0 | 2498728.0 | 4712180.0 |
| RJKB-T24 rep1 | 2549482.0 | 2971301.0 | 1474871.0 | 2787697.0 |
| RJKB-T24 rep2 | 4192231.0 | 4433169.0 | 2573304.0 | 4778009.0 |
| RJKB-T23-NV (2 DAS) | 8124571.0 | 8519668.0 | 4072384.0 | 7458267.0 |
| RJKB-T23-NV (14 DAS) rep1 | 7068465.0 | 7574954.0 | 3513082.0 | 6722707.0 |
| RJKB-T23-NV (14 DAS) rep2 | 7061660.0 | 7374104.0 | 3877172.0 | 6715249.0 |
| RJKB-T24-NV (2 DAS) | 4108931.0 | 4643563.0 | 2114726.0 | 3828520.0 |
| RJKB-T24-NV (14 DAS) rep1 | 4920545.0 | 5066318.0 | 2432701.0 | 4625054.0 |
| RJKB-T24-NV (14 DAS) rep2 | 6927559.0 | 6762614.0 | 3783487.0 | 6474412.0 |
| RJKB-T24 4wkV | 5723551.0 | 5850405.0 | 3003627.0 | 5487379.0 |
| RJKB-T24 4wkV+12d | 5612568.0 | 6032169.0 | 2897528.0 | 5320993.0 |Figure S6. Mapped reads of input and immunoprecipitated DNA using anti-H3K27me3 antibodies in genic regions classified into 2kb-upstream, exon, intron, and 2kb downstream positions.

## Slide 7
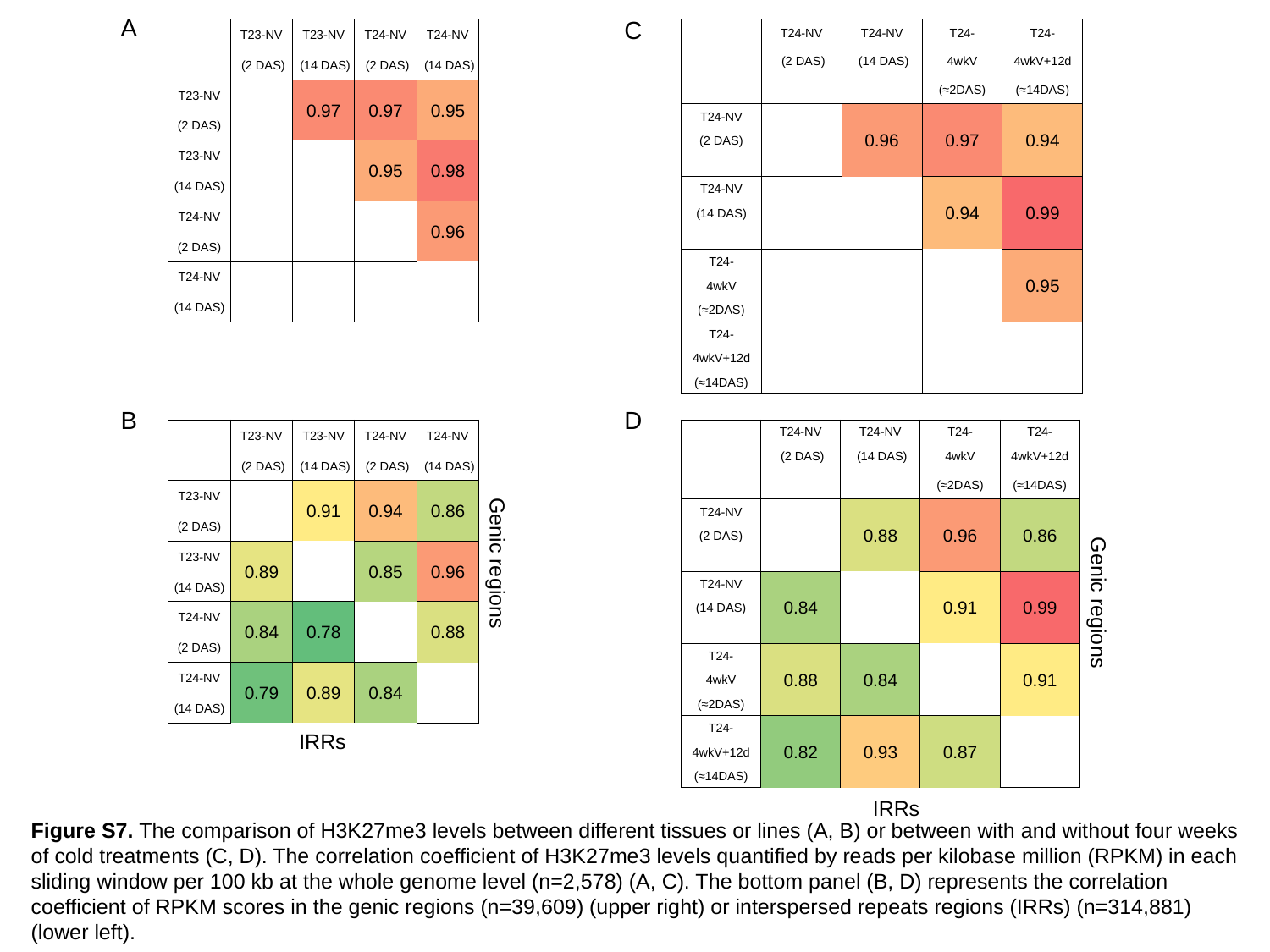

A
C
| | T23-NV | T23-NV | T24-NV | T24-NV |
| --- | --- | --- | --- | --- |
| | (2 DAS) | (14 DAS) | (2 DAS) | (14 DAS) |
| T23-NV | | 0.97 | 0.97 | 0.95 |
| (2 DAS) | | | | |
| T23-NV | | | 0.95 | 0.98 |
| (14 DAS) | | | | |
| T24-NV | | | | 0.96 |
| (2 DAS) | | | | |
| T24-NV | | | | |
| (14 DAS) | | | | |
| | T24-NV | T24-NV | T24- | T24- |
| --- | --- | --- | --- | --- |
| | (2 DAS) | (14 DAS) | 4wkV | 4wkV+12d |
| | | | (≈2DAS) | (≈14DAS) |
| T24-NV | | 0.96 | 0.97 | 0.94 |
| (2 DAS) | | | | |
| | | | | |
| T24-NV | | | 0.94 | 0.99 |
| (14 DAS) | | | | |
| | | | | |
| T24- | | | | 0.95 |
| 4wkV | | | | |
| (≈2DAS) | | | | |
| T24- | | | | |
| 4wkV+12d | | | | |
| (≈14DAS) | | | | |
B
D
| | T23-NV | T23-NV | T24-NV | T24-NV |
| --- | --- | --- | --- | --- |
| | (2 DAS) | (14 DAS) | (2 DAS) | (14 DAS) |
| T23-NV | | 0.91 | 0.94 | 0.86 |
| (2 DAS) | | | | |
| T23-NV | 0.89 | | 0.85 | 0.96 |
| (14 DAS) | | | | |
| T24-NV | 0.84 | 0.78 | | 0.88 |
| (2 DAS) | | | | |
| T24-NV | 0.79 | 0.89 | 0.84 | |
| (14 DAS) | | | | |
| | T24-NV | T24-NV | T24- | T24- |
| --- | --- | --- | --- | --- |
| | (2 DAS) | (14 DAS) | 4wkV | 4wkV+12d |
| | | | (≈2DAS) | (≈14DAS) |
| T24-NV | | 0.88 | 0.96 | 0.86 |
| (2 DAS) | | | | |
| | | | | |
| T24-NV | 0.84 | | 0.91 | 0.99 |
| (14 DAS) | | | | |
| | | | | |
| T24- | 0.88 | 0.84 | | 0.91 |
| 4wkV | | | | |
| (≈2DAS) | | | | |
| T24- | 0.82 | 0.93 | 0.87 | |
| 4wkV+12d | | | | |
| (≈14DAS) | | | | |
Genic regions
Genic regions
IRRs
IRRs
Figure S7. The comparison of H3K27me3 levels between different tissues or lines (A, B) or between with and without four weeks of cold treatments (C, D). The correlation coefficient of H3K27me3 levels quantified by reads per kilobase million (RPKM) in each sliding window per 100 kb at the whole genome level (n=2,578) (A, C). The bottom panel (B, D) represents the correlation coefficient of RPKM scores in the genic regions (n=39,609) (upper right) or interspersed repeats regions (IRRs) (n=314,881) (lower left).

## Slide 8
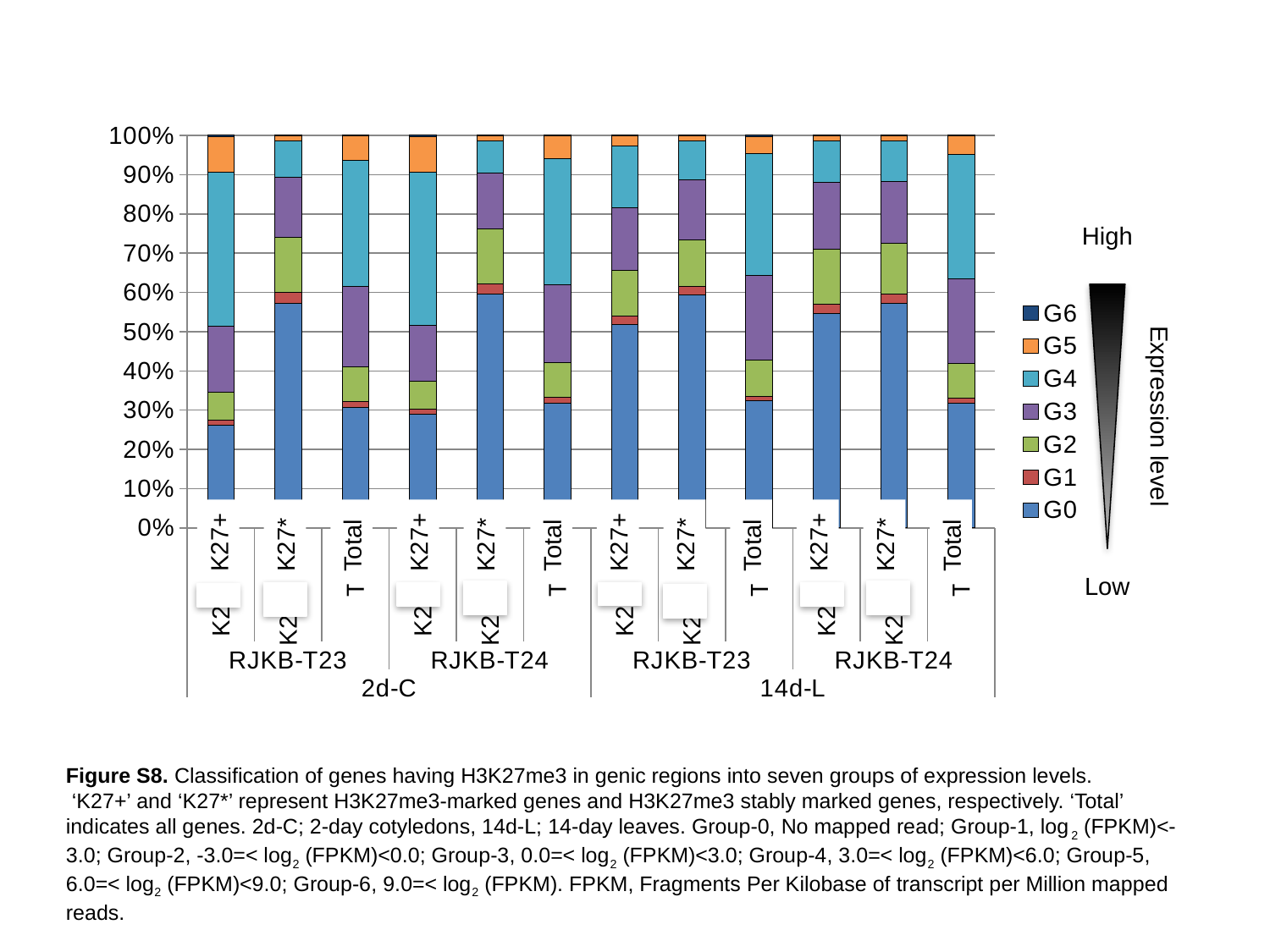

### Chart
| Category | G0 | G1 | G2 | G3 | G4 | G5 | G6 |
|---|---|---|---|---|---|---|---|
| K27me3 | 0.261840803053867 | 0.0131014656675621 | 0.0707856166643103 | 0.167114378622932 | 0.393750883642019 | 0.0905791978886846 | 0.00282765446062491 |
| K27me3* | 0.572149981062997 | 0.0279005176114127 | 0.140765054917308 | 0.151622269915415 | 0.093170054286075 | 0.0137608887766696 | 0.000631233430122459 |
| Total | 0.307662302835271 | 0.0139447573075892 | 0.0876910699919549 | 0.206270264999147 | 0.320412491772106 | 0.062458860528048 | 0.00180404202930349 |
| K27me3 | 0.288886475921603 | 0.0129757315815191 | 0.0710679189966882 | 0.142841630924589 | 0.390792116835876 | 0.0908301210706336 | 0.0026060046690917 |
| K27me3* | 0.596263098093675 | 0.0258805706350208 | 0.139628834743088 | 0.141143794975382 | 0.0835753061482136 | 0.0126246686024492 | 0.000883726802171443 |
| Total | 0.318169628708647 | 0.0135790731124601 | 0.0891294278261293 | 0.198834686364855 | 0.320924449645286 | 0.0578024817767376 | 0.00180404202930349 |
| K27me3 | 0.518446914773643 | 0.0204607700982761 | 0.117045271467698 | 0.159739004349927 | 0.157564040599323 | 0.0253745770903818 | 0.00136942162075076 |
| K27me3* | 0.592854437571014 | 0.0218406766822371 | 0.119303118293145 | 0.152253503345537 | 0.100113622017422 | 0.0128771619744982 | 0.000757480116146951 |
| Total | 0.323825544259977 | 0.0122138521173115 | 0.0908847119627489 | 0.217289548745703 | 0.309490723810917 | 0.0442234086642775 | 0.00231599990248421 |
| K27me3 | 0.545173479903813 | 0.0249914118859498 | 0.139213328753006 | 0.171934043284095 | 0.104860872552387 | 0.0127962899347303 | 0.00103057368601855 |
| K27me3* | 0.572528721121071 | 0.023103143542482 | 0.129402853175104 | 0.15768211084459 | 0.103774775912132 | 0.0124984219164247 | 0.00100997348819593 |
| Total | 0.317633291889124 | 0.0132621468100149 | 0.0877398278846388 | 0.215314854092006 | 0.31870596552817 | 0.045613008605768 | 0.00197469465369707 |High
Expression level
Low
K27+
K27*
Total
K27+
K27*
Total
K27+
K27*
Total
K27+
K27*
Total
Figure S8. Classification of genes having H3K27me3 in genic regions into seven groups of expression levels.
 ‘K27+’ and ‘K27*’ represent H3K27me3-marked genes and H3K27me3 stably marked genes, respectively. ‘Total’ indicates all genes. 2d-C; 2-day cotyledons, 14d-L; 14-day leaves. Group-0, No mapped read; Group-1, log2 (FPKM)<-3.0; Group-2, -3.0=< log2 (FPKM)<0.0; Group-3, 0.0=< log2 (FPKM)<3.0; Group-4, 3.0=< log2 (FPKM)<6.0; Group-5, 6.0=< log2 (FPKM)<9.0; Group-6, 9.0=< log2 (FPKM). FPKM, Fragments Per Kilobase of transcript per Million mapped reads.

## Slide 9
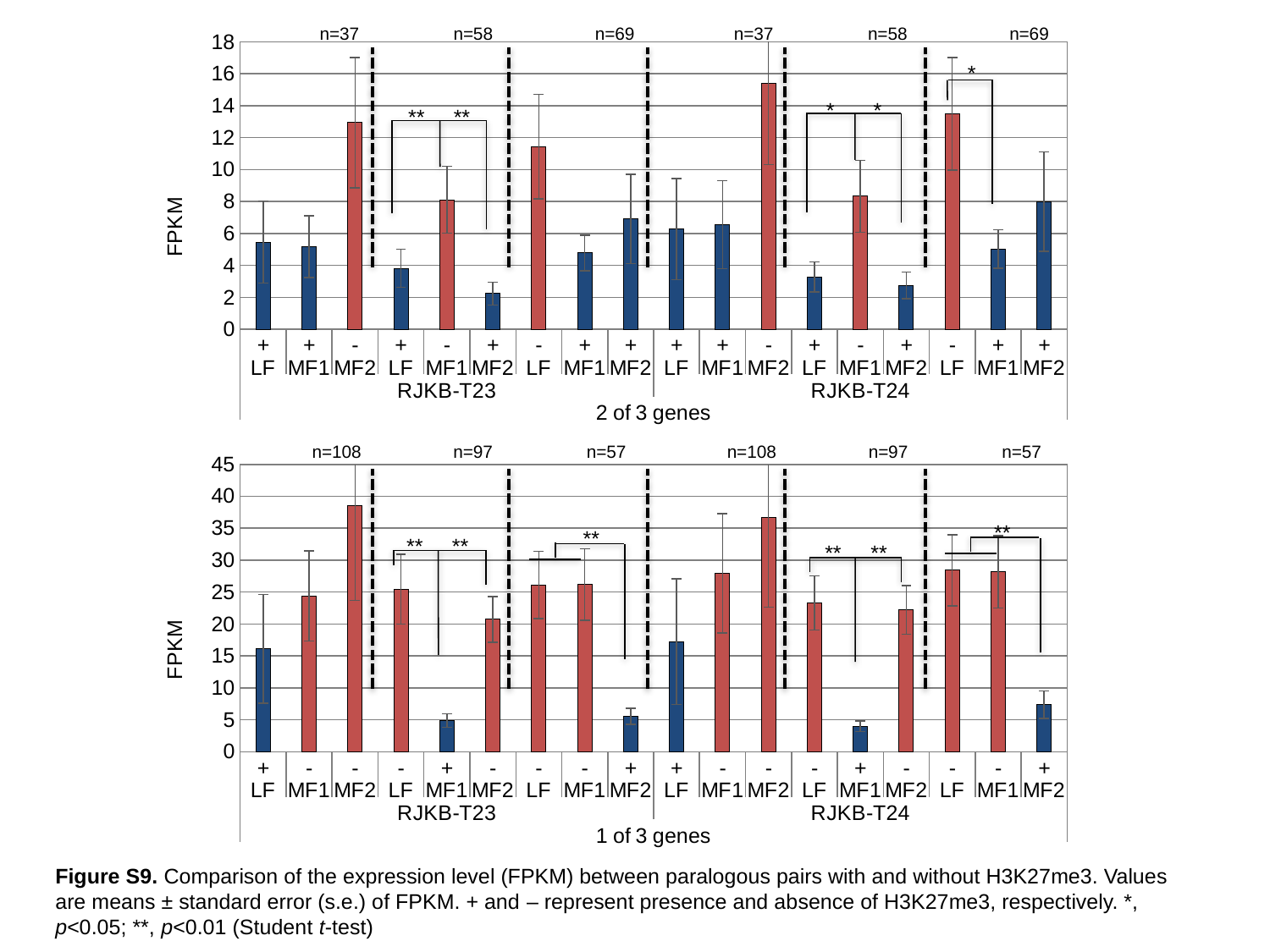

n=37
n=58
n=69
n=37
n=58
n=69
### Chart
| Category | |
|---|---|
| + | 5.455500189189187 |
| + | 5.168716094594592 |
| - | 12.93874547567568 |
| + | 3.816212120689635 |
| - | 8.11080295 |
| + | 2.231498896551724 |
| - | 11.43794627971014 |
| + | 4.770154539130424 |
| + | 6.91140247536232 |
| + | 6.27077627027027 |
| + | 6.54493795313792 |
| - | 15.37429873569241 |
| + | 3.28330770689655 |
| - | 8.328326764895843 |
| + | 2.736286968780749 |
| - | 13.49906234782608 |
| + | 5.015916869565173 |
| + | 7.98998170289855 |*
*
*
**
**
n=108
n=97
n=57
n=108
n=97
n=57
### Chart
| Category | |
|---|---|
| + | 16.08563670877172 |
| - | 24.40306305964912 |
| - | 38.52898012280702 |
| - | 25.43366417525775 |
| + | 4.876754525773194 |
| - | 20.72810245876288 |
| - | 26.13725603703704 |
| - | 26.17326632407407 |
| + | 5.521215401851852 |
| + | 17.24356186666667 |
| - | 27.92130785964912 |
| - | 36.6407404385965 |
| - | 23.31302307216495 |
| + | 3.967674344329898 |
| - | 22.21939348659794 |
| - | 28.40911946296292 |
| - | 28.17079750925926 |
| + | 7.32569320185185 |**
**
**
**
**
**
Figure S9. Comparison of the expression level (FPKM) between paralogous pairs with and without H3K27me3. Values are means ± standard error (s.e.) of FPKM. + and – represent presence and absence of H3K27me3, respectively. *, p<0.05; **, p<0.01 (Student t-test)

## Slide 10
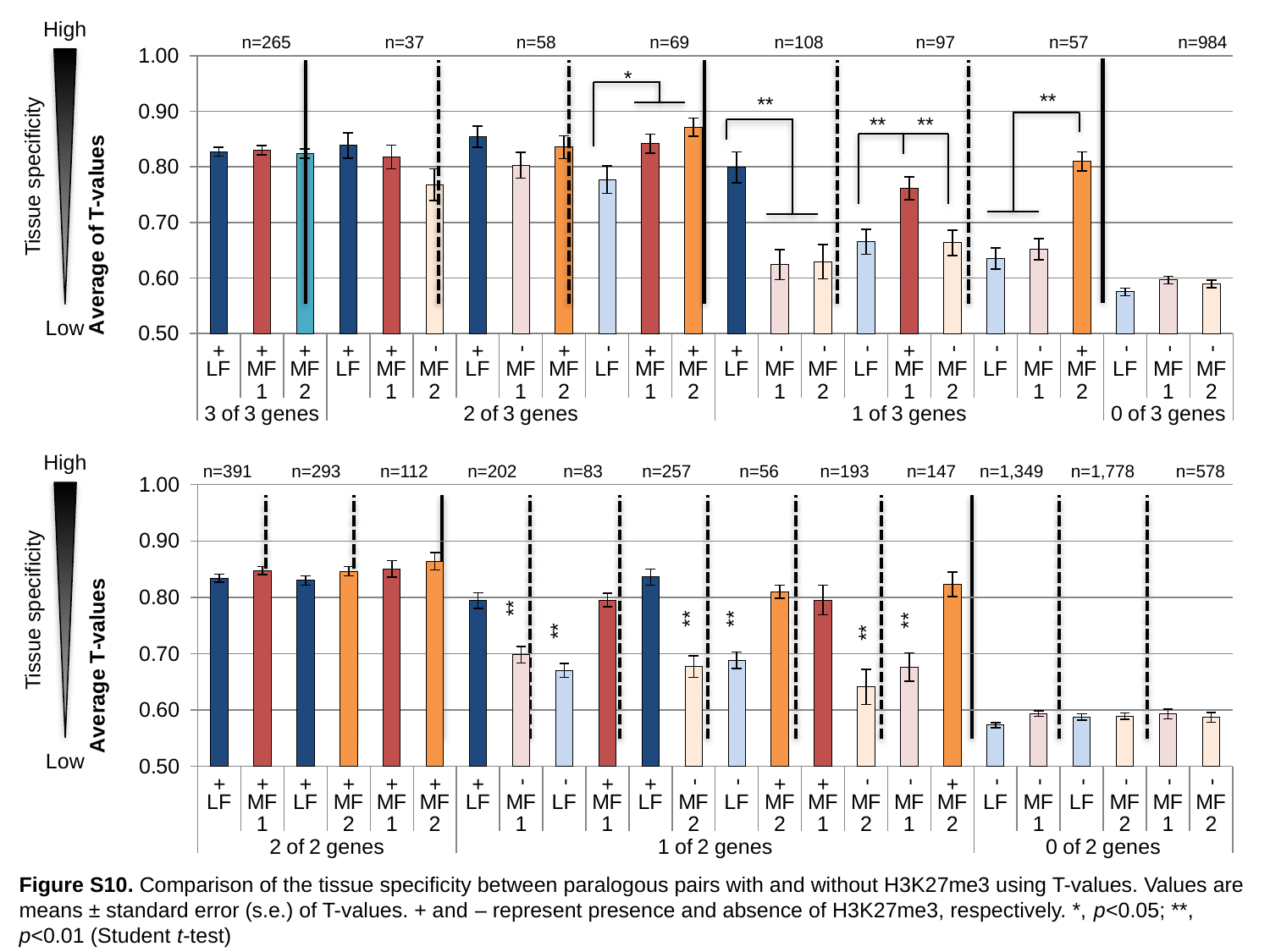

High
Tissue specificity
Low
### Chart
| Category | |
|---|---|
| + | 0.827270036872105 |
| + | 0.83004163858669 |
| + | 0.824064305747207 |
| + | 0.83849960137071 |
| + | 0.81779071917531 |
| - | 0.76794793922948 |
| + | 0.854568530220167 |
| - | 0.80295032189562 |
| + | 0.835424470020988 |
| - | 0.777010647993764 |
| + | 0.841587010627782 |
| + | 0.871838317674571 |
| + | 0.799067958815284 |
| - | 0.62356354100432 |
| - | 0.629035440652773 |
| - | 0.664804109784911 |
| + | 0.761463414634146 |
| - | 0.663152173913043 |
| - | 0.634856494575254 |
| - | 0.651745334834942 |
| + | 0.810013745787647 |
| - | 0.574715583684878 |
| - | 0.596135427816462 |
| - | 0.589210953592998 |*
**
**
**
**
High
Tissue specificity
Low
### Chart
| Category | |
|---|---|
| + | 0.834060409159891 |
| + | 0.847432432432433 |
| + | 0.829967406958489 |
| + | 0.846383763837638 |
| + | 0.850653371832262 |
| + | 0.863917525773196 |
| + | 0.794359581822722 |
| - | 0.697932960893855 |
| - | 0.669908238529873 |
| + | 0.795 |
| + | 0.835925925925926 |
| - | 0.677028985507247 |
| - | 0.688248815693693 |
| + | 0.810117647058823 |
| + | 0.795154387159078 |
| - | 0.640862357732859 |
| - | 0.676278966321598 |
| + | 0.822876712328767 |
| - | 0.573086692808585 |
| - | 0.593420745920746 |
| - | 0.587891373187058 |
| - | 0.588926123381568 |
| - | 0.593063127685403 |
| - | 0.587016274864377 |**
**
**
**
**
**
n=265
n=37
n=58
n=69
n=108
n=97
n=57
n=984
n=391
n=293
n=112
n=202
n=83
n=257
n=56
n=193
n=147
n=1,349
n=1,778
n=578
Figure S10. Comparison of the tissue specificity between paralogous pairs with and without H3K27me3 using T-values. Values are means ± standard error (s.e.) of T-values. + and – represent presence and absence of H3K27me3, respectively. *, p<0.05; **, p<0.01 (Student t-test)

## Slide 11
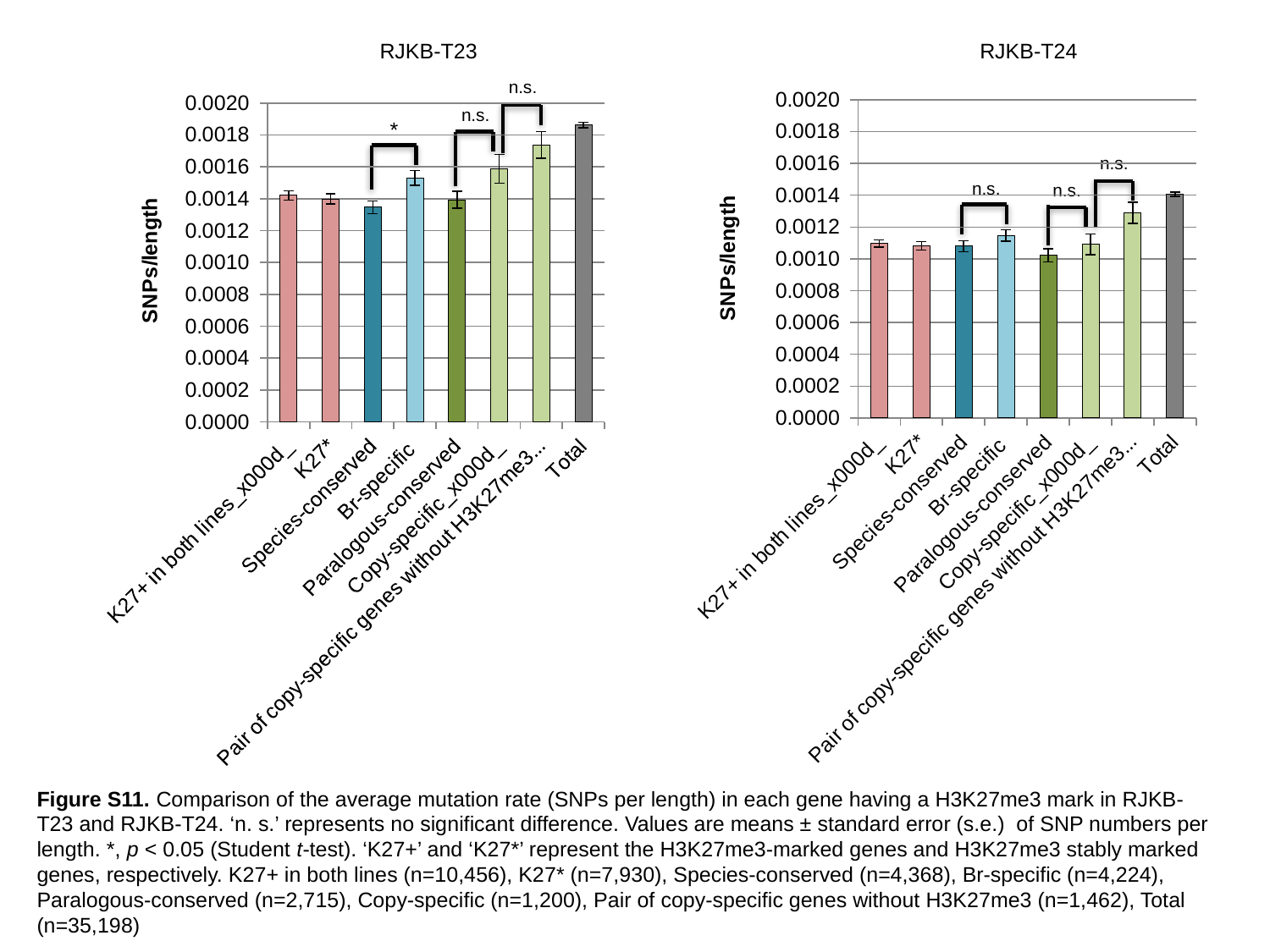

RJKB-T23
RJKB-T24
n.s.
### Chart
| Category | |
|---|---|
| K27+ in both lines_x000d_ | 0.00142033424086915 |
| K27* | 0.00139851915440982 |
| Species-conserved | 0.00134625880466996 |
| Br-specific | 0.00153004411863468 |
| Paralogous-conserved | 0.00139242943994022 |
| Copy-specific_x000d_ | 0.00158799043951139 |
| Pair of copy-specific genes without H3K27me3_x000d_ | 0.00173727071559064 |
| Total | 0.00186145666514668 |
### Chart
| Category | |
|---|---|
| K27+ in both lines_x000d_ | 0.00109517195450468 |
| K27* | 0.00108209613086249 |
| Species-conserved | 0.00107970897442285 |
| Br-specific | 0.00114718656891097 |
| Paralogous-conserved | 0.00102232057367297 |
| Copy-specific_x000d_ | 0.00109188976329109 |
| Pair of copy-specific genes without H3K27me3_x000d_ | 0.00129033015600628 |
| Total | 0.00140576378508244 |n.s.
*
n.s.
n.s.
n.s.
Figure S11. Comparison of the average mutation rate (SNPs per length) in each gene having a H3K27me3 mark in RJKB-T23 and RJKB-T24. ‘n. s.’ represents no significant difference. Values are means ± standard error (s.e.) of SNP numbers per length. *, p < 0.05 (Student t-test). ‘K27+’ and ‘K27*’ represent the H3K27me3-marked genes and H3K27me3 stably marked genes, respectively. K27+ in both lines (n=10,456), K27* (n=7,930), Species-conserved (n=4,368), Br-specific (n=4,224), Paralogous-conserved (n=2,715), Copy-specific (n=1,200), Pair of copy-specific genes without H3K27me3 (n=1,462), Total (n=35,198)

## Slide 12
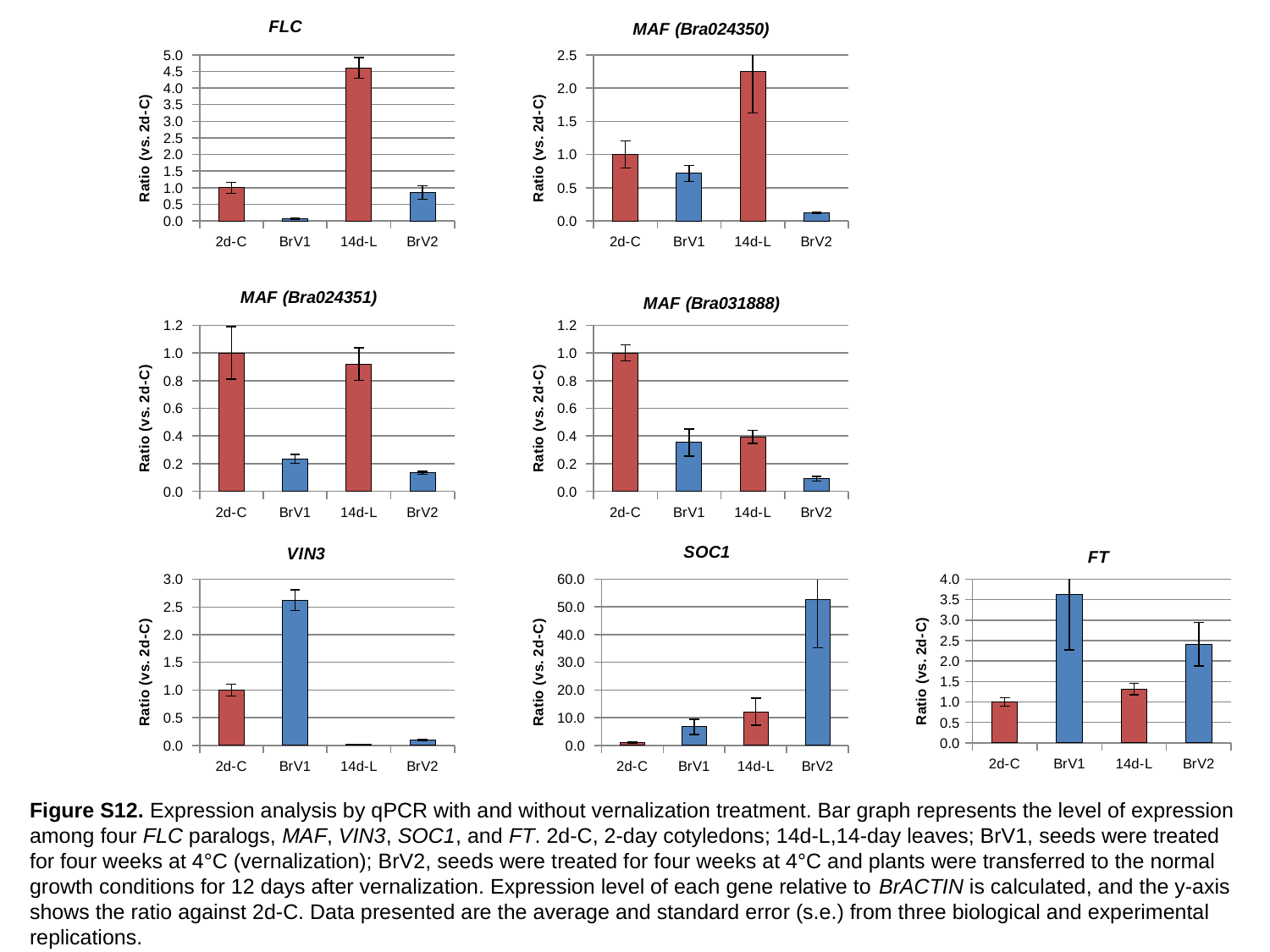

### Chart: FLC
| Category | |
|---|---|
| 2d-C | 1.0 |
| BrV1 | 0.0758356000076067 |
| 14d-L | 4.6075010637621 |
| BrV2 | 0.860720575137489 |
### Chart: MAF (Bra024350)
| Category | |
|---|---|
| 2d-C | 1.0 |
| BrV1 | 0.716117626583681 |
| 14d-L | 2.243058878104793 |
| BrV2 | 0.128748899375129 |
### Chart: MAF (Bra024351)
| Category | |
|---|---|
| 2d-C | 1.0 |
| BrV1 | 0.234097992606147 |
| 14d-L | 0.918352108971107 |
| BrV2 | 0.133173411598001 |
### Chart: MAF (Bra031888)
| Category | |
|---|---|
| 2d-C | 1.0 |
| BrV1 | 0.352344581978298 |
| 14d-L | 0.393464260259422 |
| BrV2 | 0.0918234974785229 |
### Chart: VIN3
| Category | |
|---|---|
| 2d-C | 1.0 |
| BrV1 | 2.62449030831154 |
| 14d-L | 0.0147439274385126 |
| BrV2 | 0.100048505260083 |
### Chart: SOC1
| Category | |
|---|---|
| 2d-C | 1.0 |
| BrV1 | 6.676834641094344 |
| 14d-L | 12.14180398074483 |
| BrV2 | 52.51734280498384 |
### Chart: FT
| Category | |
|---|---|
| 2d-C | 1.0 |
| BrV1 | 3.614750114394277 |
| 14d-L | 1.313194166116558 |
| BrV2 | 2.408122088032831 |Figure S12. Expression analysis by qPCR with and without vernalization treatment. Bar graph represents the level of expression among four FLC paralogs, MAF, VIN3, SOC1, and FT. 2d-C, 2-day cotyledons; 14d-L,14-day leaves; BrV1, seeds were treated for four weeks at 4°C (vernalization); BrV2, seeds were treated for four weeks at 4°C and plants were transferred to the normal growth conditions for 12 days after vernalization. Expression level of each gene relative to BrACTIN is calculated, and the y-axis shows the ratio against 2d-C. Data presented are the average and standard error (s.e.) from three biological and experimental replications.

## Slide 13
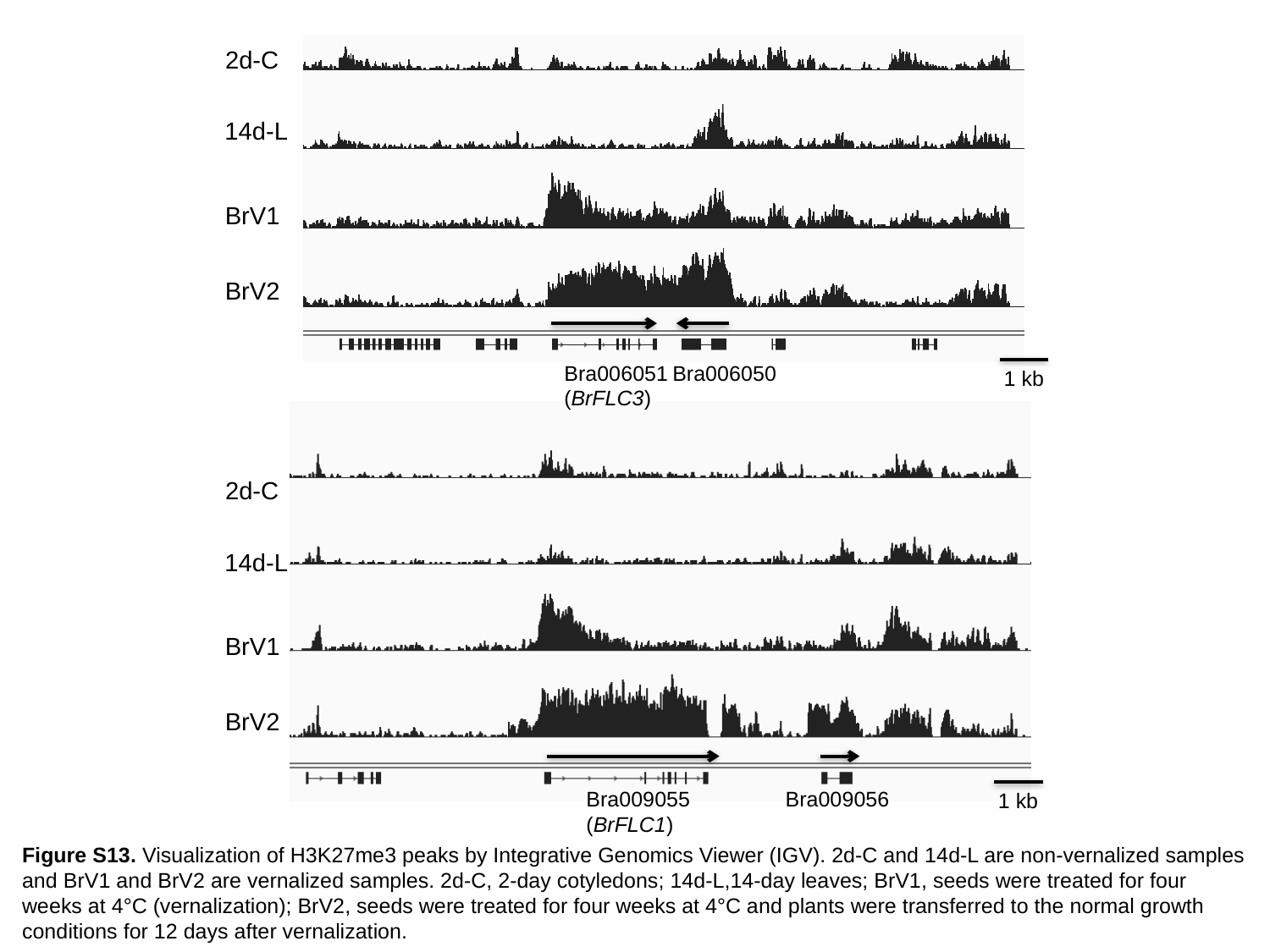

2d-C
14d-L
BrV1
BrV2
Bra006051
(BrFLC3)
Bra006050
1 kb
2d-C
14d-L
BrV1
BrV2
Bra009055
(BrFLC1)
Bra009056
1 kb
Figure S13. Visualization of H3K27me3 peaks by Integrative Genomics Viewer (IGV). 2d-C and 14d-L are non-vernalized samples and BrV1 and BrV2 are vernalized samples. 2d-C, 2-day cotyledons; 14d-L,14-day leaves; BrV1, seeds were treated for four weeks at 4°C (vernalization); BrV2, seeds were treated for four weeks at 4°C and plants were transferred to the normal growth conditions for 12 days after vernalization.

## Slide 14
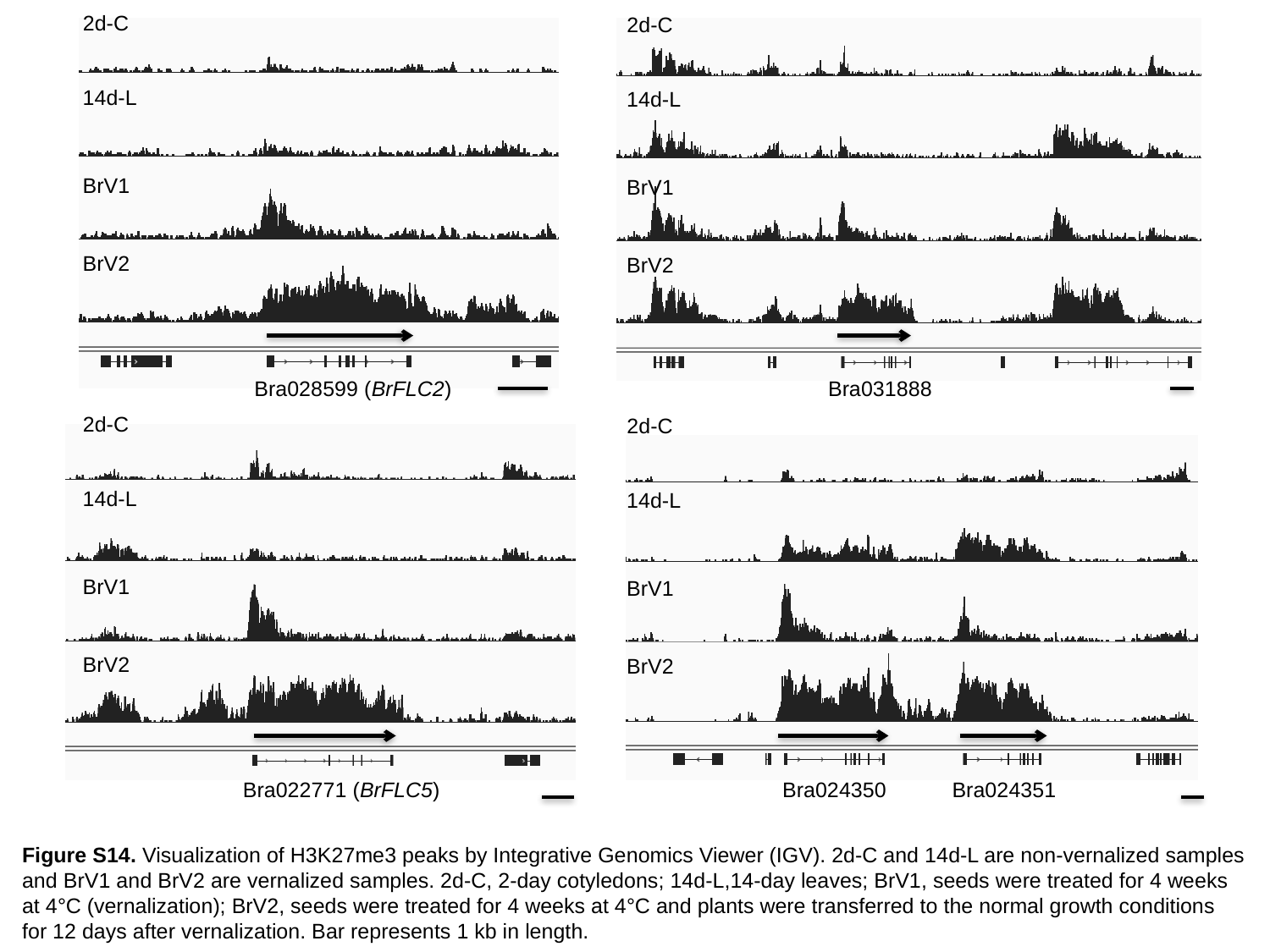

2d-C
2d-C
14d-L
14d-L
BrV1
BrV1
BrV2
BrV2
Bra028599 (BrFLC2)
Bra031888
2d-C
2d-C
14d-L
14d-L
BrV1
BrV1
BrV2
BrV2
Bra022771 (BrFLC5)
Bra024350
Bra024351
Figure S14. Visualization of H3K27me3 peaks by Integrative Genomics Viewer (IGV). 2d-C and 14d-L are non-vernalized samples and BrV1 and BrV2 are vernalized samples. 2d-C, 2-day cotyledons; 14d-L,14-day leaves; BrV1, seeds were treated for 4 weeks at 4°C (vernalization); BrV2, seeds were treated for 4 weeks at 4°C and plants were transferred to the normal growth conditions for 12 days after vernalization. Bar represents 1 kb in length.

## Slide 15
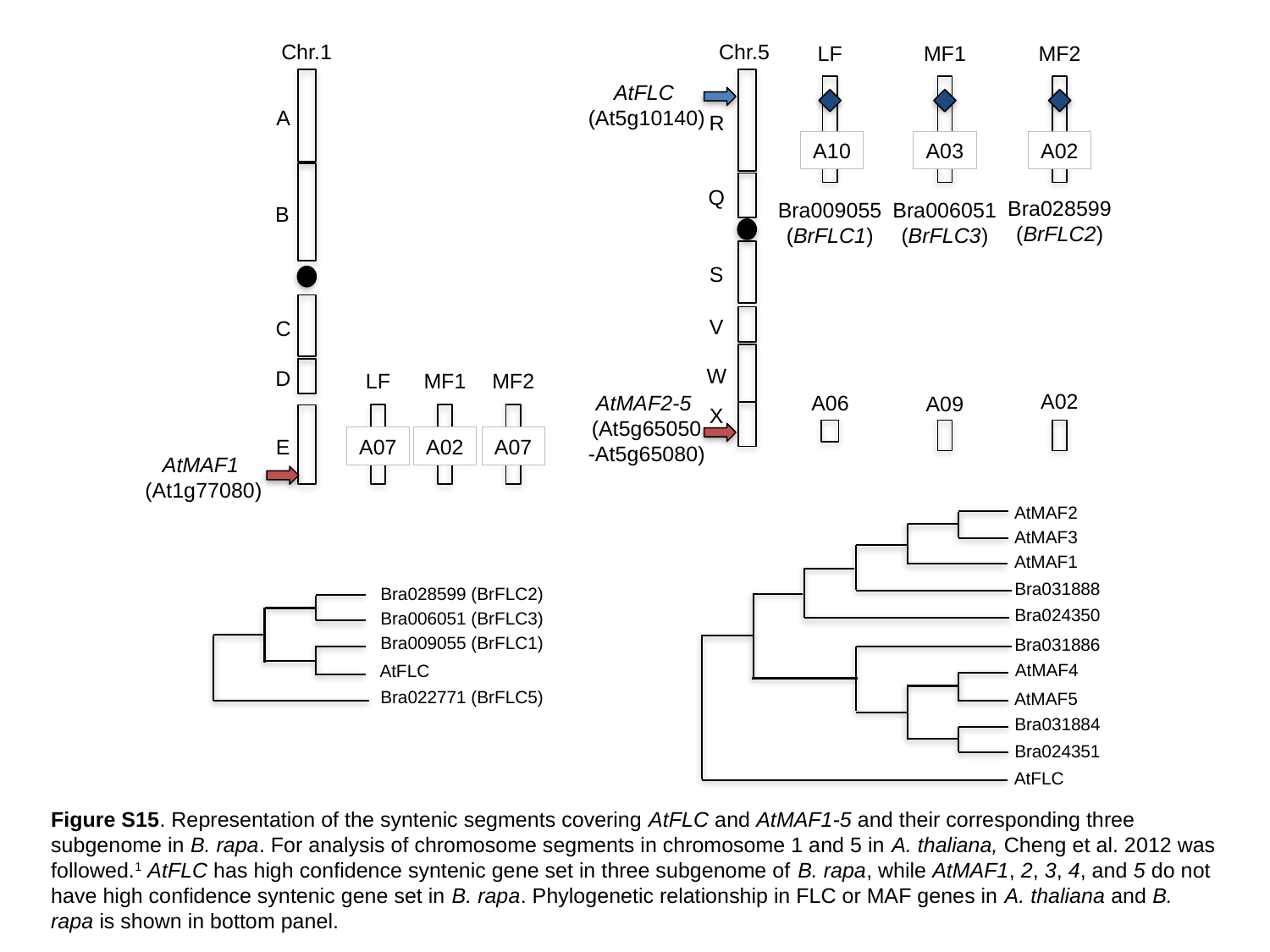

Chr.1
Chr.5
LF
A10
Bra009055
(BrFLC1)
A06
MF1
A03
Bra006051
(BrFLC3)
A09
MF2
A02
Bra028599
(BrFLC2)
A02
AtFLC
(At5g10140)
A
R
Q
B
S
V
C
W
D
LF
A07
MF1
A02
MF2
A07
AtMAF2-5
(At5g65050
-At5g65080)
X
E
AtMAF1
(At1g77080)
AtMAF2
AtMAF3
AtMAF1
Bra031888
Bra024350
Bra031886
AtMAF4
AtMAF5
Bra031884
Bra024351
AtFLC
Bra028599 (BrFLC2)
Bra006051 (BrFLC3)
Bra009055 (BrFLC1)
AtFLC
Bra022771 (BrFLC5)
Figure S15. Representation of the syntenic segments covering AtFLC and AtMAF1-5 and their corresponding three subgenome in B. rapa. For analysis of chromosome segments in chromosome 1 and 5 in A. thaliana, Cheng et al. 2012 was followed.1 AtFLC has high confidence syntenic gene set in three subgenome of B. rapa, while AtMAF1, 2, 3, 4, and 5 do not have high confidence syntenic gene set in B. rapa. Phylogenetic relationship in FLC or MAF genes in A. thaliana and B. rapa is shown in bottom panel.
